# Genome-Wide Identification of Effector Candidates With Conserved Motifs From the Wheat Leaf Rust Fungus *Puccinia triticina*

**DOI:** 10.3389/fmicb.2020.01188

**Published:** 2020-06-03

**Authors:** Shuqing Zhao, Xiaofeng Shang, Weishuai Bi, Xiumei Yu, Daqun Liu, Zhensheng Kang, Xiaojie Wang, Xiaodong Wang

**Affiliations:** ^1^State Key Laboratory of North China Crop Improvement and Regulation, College of Plant Protection, Technological Innovation Center for Biological Control of Crop Diseases and Insect Pests of Hebei Province, Hebei Agricultural University, Baoding, China; ^2^State Key Laboratory of Crop Stress Biology for Arid Areas, College of Plant Protection, Northwest A&F University, Xianyang, China; ^3^College of Life Sciences, Hebei Agricultural University, Baoding, China

**Keywords:** fungal effectors, conserved motif, wheat leaf rust, transcriptome, RXLR, CRN, Y/F/WxC, CFEM

## Abstract

Rust fungi secrete various specialized effectors into host cells to manipulate the plant defense response. Conserved motifs, including RXLR, LFLAK-HVLVxxP (CRN), Y/F/WxC, CFEM, LysM, EAR, [SG]-P-C-[KR]-P, DPBB_1 (PNPi), and ToxA, have been identified in various oomycete and fungal effectors and are reported to be crucial for effector translocation or function. However, little is known about potential effectors containing any of these conserved motifs in the wheat leaf rust fungus (*Puccinia triticina*, *Pt*). In this study, sequencing was performed on RNA samples collected from the germ tubes (GT) of uredospores of an epidemic *Pt* pathotype PHTT(P) and *Pt*-infected leaves of a susceptible wheat cultivar “Chinese Spring” at 4, 6, and 8 days post-inoculation (dpi). The assembled transcriptome data were compared to the reference genome of “*Pt* 1-1 BBBD Race 1.” A total of 17,976 genes, including 2,284 “novel” transcripts, were annotated. Among all these genes, we identified 3,149 upregulated genes upon *Pt* infection at all time points compared to GT, whereas 1,613 genes were more highly expressed in GT. A total of 464 secreted proteins were encoded by those upregulated genes, with 79 of them also predicted as possible effectors by EffectorP. Using hmmsearch and Regex, we identified 719 RXLR-like, 19 PNPi-like, 19 CRN-like, 138 Y/F/WxC, and 9 CFEM effector candidates from the deduced protein database including data based on the “*Pt* 1-1 BBBD Race 1” genome and the transcriptome data collected here. Four of the PNPi-like effector candidates with DPBB_1 conserved domain showed physical interactions with wheat NPR1 protein in yeast two-hybrid assay. Nine Y/F/WxC and seven CFEM effector candidates were transiently expressed in *Nicotiana benthamiana*. None of these effector candidates showed induction or suppression of cell death triggered by BAX protein, but the expression of one CFEM effector candidate, PTTG_08198, accelerated the progress of cell death and promoted the accumulation of reactive oxygen species (ROS). In conclusion, we profiled genes associated with the infection process of the *Pt* pathotype PHTT(P). The identified effector candidates with conserved motifs will help guide the investigation of virulent mechanisms of leaf rust fungus.

## Introduction

Wheat leaf rust, caused by *Puccinia triticina* (*Pt*), has expanded its epidemic region to most of the wheat-cultivating area in China due to changes associated with global warming and high-density wheat planting (Helfer, 2014). The yield reduction caused by this disease ranges from 5 to 20%, and can reach as high as 50% during epidemics (Eversmeyer and Kramer, 2000; Bolton et al., 2008). The successful biotrophic lifestyle of rust fungi relies on the ability to secrete various effectors into the inter-cellular space, some of which further translocate into plant cells and act to suppress or evade plant defense response.

To understand the virulent mechanism of wheat rust species, genomic and transcriptomic sequencing has been applied to wheat stripe rust (*Puccinia striiformis* f. sp. *tritici*, *Pst*), stem rust (*Puccinia graminis* f. sp. *tritici*, *Pgt*), and leaf rust (Cantu et al., 2011; Duplessis et al., 2011; Zheng et al., 2013; Kiran et al., 2016; Xu et al., 2020). The draft genome of the wheat leaf rust fungus has greatly facilitated efforts to identify genes associated with leaf rust infection (Kiran et al., 2016). For example, the effective secretome of leaf rust fungus was identified by sequencing RNA samples collected from wheat leaves infected with six *Pt* pathotypes at 6 days post-inoculation (dpi) (Bruce et al., 2014). In a separate study, comparative genomic analysis was used to initially explore candidate effector genes corresponding to the wheat leaf rust resistance gene *Lr20* (Wu et al., 2017). Recent progress in dissecting high quality dikaryotic genomes of the wheat leaf rust fungus revealed the natural somatic exchange mechanisms of the pathogen (Wu et al., 2019). However, the expression levels of genes in the germ tube and during early infection stages of *Pt* uredospores have not been profiled, and a time-resolved transcriptional investigation is needed to identify specific genes associated with the *Pt* infection process.

Using available genomic resources and specialized transcriptome sequencing, various rust effectors have been identified and initially characterized during the last decade (Lorrain et al., 2018). The bioinformatics program EffectorP uses machine learning to predict effector candidates from the fungal secretome (Sperschneider et al., 2016). Additionally, tools such as yeast two-hybrid (Y2H) library screening and immunoprecipitation of GFP-fused effectors coupled with liquid chromatography–tandem mass spectrometry (CoIP/MS) have been used to confirm wheat targets of several rust effectors. The wheat stripe rust effector PEC6 was reported to suppress pattern-triggered immunity (PTI) by physically interacting with wheat adenosine kinases (Liu et al., 2016). Another effector from *Pst*, PST02549, showed protein interaction with wheat enhancer of mRNA decapping protein 4 (EDC4) and exhibited association with processing bodies (Petre et al., 2016b). A haustorium-specific *Pst* effector Pst_12806 is translocated into wheat chloroplasts to target wheat cytochrome b6-f complex TaISP protein and suppress plant basal immunity (Xu et al., 2019). Another glycine-serine-rich effector from wheat stripe rust, PstGSRE1, decreases the accumulation of reactive oxygen species (ROS) by targeting the transcription factor TaLOL2 (Qi et al., 2019).

In a previous study, we found that a virulent *Pst* effector, PNPi, directly interacted with wheat NPR1 (wNPR1) protein and suppressed the wNPR1-mediated systemic acquired resistance (Wang et al., 2016). A string of amino acids (RSLL–DEEP) at the N-terminus of the PNPi is located after the cleavage site of the predicted signal peptide, and is similar to the RXLR motif (PF16810) frequently detected in oomycete effectors. The RXLR motif may guide the translocation of *Phytophthora sojae* effectors into plant cells (Kale et al., 2010). Other studies suggested that re-entry signals of certain RXLR effectors (AvrM and AVR3a) might be triggered by traffic congestion of the secretion pathway, and the cleavage of the RXLR motif of AVR3a occurred before secretion (Petre et al., 2016a; Wawra et al., 2017). Another investigation of animal-pathogenic oomycete *Saprolegnia parasitica* found that the uptake process of a host-targeting protein SpHtp3 is guided by a gp96-like receptor via its C-terminal YKARK region, but not by the N-terminal RXLR motif (Trusch et al., 2018).

In addition to the RXLR motif in oomycete effectors, several other conserved motifs, including LFLAK-HVLVXXP motif in oomycete CRN effectors, Y/F/WxC motif in powdery mildew effectors, and [SG]-P-C-[KR]-P motif in *Fusarium* effectors, were also predicted to facilitate effector up-take processes (Godfrey et al., 2010; Schornack et al., 2010; Sperschneider et al., 2013). Other conserved motifs identified from well-characterized effectors may be more functionally specialized. For example, fungal effectors containing LysM motif (PF01476) act in both suppressing chitin-triggered immune response and regulation of fungal development (De Jonge et al., 2010; Seidl-Seiboth et al., 2013). The EAR motif (PF07897) was shown to be required for avirulence of effector protein PopP2 from *Ralstonia solanacearum*, possibly by recruitment of transcriptional co-repressors (Cécile et al., 2017). The Toxin_ToxA motif (PF11584) from *Pyrenophora tritici-repentis* proteinaceous host-selective toxin ToxA is necessary and sufficient to induce cell death in sensitive wheat cultivars (Sarma et al., 2005).

In this study, a transcriptome sequencing approach was used to analyze RNA samples collected from germinated urediospores of virulent *Pt* pathotype PHTT(P) and infected wheat leaves at 4, 6, and 8 dpi, allowing the identification of DEGs associated with the *Pt* infection process. A total of 79 effector candidates encoded by the upregulated DEGs were predicted using SignalP and EffectorP. From the deduced protein database of the “*Pt* 1-1 BBBD Race 1” genome and the transcriptome data, effector candidates with conserved motifs were initially identified by hmmsearch, Regex, and homology analysis. Several differentially expressed effector candidates containing these conserved motifs were selected for further functional characterization.

## Materials and Methods

### Rust Inoculation and RNA Samples

Uredospores of the epidemic *Pt* pathotype PHTT(P) were collected from the field as described in our earlier study (Zhang et al., 2020). Seedling plants of the common wheat cultivar “Chinese Spring” were grown in a glasshouse. The fully expanded third leaves of the wheat seedling plants were spray-inoculated with uredospores of *Pt* pathotype PHTT(P) in water solution. RNA samples were collected from the *Pt*-inoculated leaves at 4, 6, and 8 dpi. The uredospores of the same *Pt* pathotype were germinated overnight and sampled to serve as a control. We used three independent biological replicates to generate samples for RNA-seq assay, and four replicates were included in the qRT-PCR assay. Harvested samples were immediately transferred into liquid nitrogen.

### Transcriptome Sequencing

RNA was isolated using an RNA extraction kit (QIAGEN, Hilden, Germany). KAPA library preparation and Illumina sequencing were conducted on a Novaseq 6000 System by Novogene Co., Ltd. The published genome of “*Pt* 1-1 BBBD Race 1” (Kiran et al., 2016) was employed as the reference for the assembly of the transcriptome using Hisat2 v2.0.5 (Kim et al., 2015). StringTie was utilized to compare all the sequenced reads with gene models in the reference genome (Pertea et al., 2015). Clusters that could not be found in the reference genome (class_code “u”) were designated as “novel” transcripts. Open reading frames (ORFs) of the “novel” transcripts were predicted using glimmer v3.02, and the deduced protein sequences were combined with the protein database from “*Pt* 1-1 BBBD Race 1” genome and subjected to hmmsearch, Regex, and homology analysis. Since the Person’s correlations of overall gene expression levels in “GT_3 (Sample #3 from germ tubes)” and “4_dpi_3 (Sample #3 from 4 days post-inoculation)” with their corresponding biological replicates were less than 0.92, these two samples were excluded for further gene expression analysis. The expected number of fragments per kilobase of transcript sequence per millions base pairs (FPKM) values for each of the extracted transcripts were determined using featureCounts v1.5.0-p3 (Liao et al., 2014). By comparing the expression levels of genes among different groups with “FDR-adjusted *p*-value < 0.05” and “| Log2FoldChange| > 1,” differentially expressed genes (DEGs) were profiled using DESeq2 (Love et al., 2014). Effector candidates were predicted from 3,149 co-upregulated DEGs using SignalP^1^ and EffectorP^2^ (Supplementary Figure S1A). Conserved domains in each of the identified effectors were predicted using pfam^3^. The GOseq package was employed to assign gene ontology (GO) annotations to each of the genes (Young et al., 2010). Heatmaps were generated based on the FPKM values for each of the selected genes using MeV v4.9.0 (Howe et al., 2011). Neighbor-joining trees were constructed based on multiple sequence alignment according to the MUSCLE method using MEGA v7.0 (Edgar, 2004; Kumar et al., 2008).

### Effector Candidates With Conserved Motifs

The combined protein database was searched to identify effector candidates with conserved motifs of RXLR, DPBB_1 (PNPi), Y/F/WxC, CFEM, LysM, EAR, LFLAK-HVLVxxP (CRN), [SG]-P-C-[KR]-P, and ToxA using a customized workflow (Supplementary Figure S1B). Briefly, HMM features of RXLR (PF16810), DPBB_1 (PF03330), CFEM (PF05730), LysM (PF01476), EAR (PF07897), and ToxA (PF11584) were downloaded from the pfam website^3^. Conserved regions from 141 oomycete CRN effectors (Stam et al., 2013), 54 barley powdery mildew Y/F/WxC effectors (Godfrey et al., 2010), and 30 *Fusarium* [SG]-P-C-[KR]-P effectors (Sperschneider et al., 2013) were utilized to generate HMM features using hmmbuild. The hidden Markov models for these conserved motifs were visualized using Weblogo^4^ (Supplementary Figure S2). The combined protein database was initially searched using hmmsearch to identify proteins containing any of the previously identified conserved motifs. For RXLR-like motif, previous study indicated that substitutions of the first R with K or H, the L with I, M, F, Y, W, or K, and the fourth R with A, L, Q, G, T, or F, would still allow the translocation function of this motif (Kale et al., 2010). Regex code of (^∧^w{10,40}\w{1,96}[RKH]\w[LIMFYWK][RALQGTF]) modified from a previous study (Haas et al., 2009) was applied to identify proteins containing the RXLR-like motif from the combined protein database. All the collected RXLR-like effector candidates were further screened with Regex code of [ED][ED][KR] derived from a previous study (Haas et al., 2009) to discover proteins containing a complete RXLR-dEER-like motif. Using a similar approach, proteins containing motifs of LFLAK (CRN), [SG]-P-C-[KR]-P, and Y/F/WxC in the N-terminal region of the protein were identified using Regex codes of (^∧^\w{10,40}\w{1,96}L[FYRL][LKF][ATVRK][KRN]), (^∧^\w{10,40}\w{1,96}[GS]PC[KR]P), and (^∧^\w{10,40}\w{1,30} [YFW]\wC), respectively. Homology analysis was performed using sequences of previously published rust effectors with conserved motifs (PNPi, PtY/F/WxC, and PgtY/F/WxC) by local Blastp. Secreted proteins were identified using SignalP and the effector probability for each of the secreted protein was evaluated using EffectorP. The expression patterns of all effector candidates were profiled based on their FPKM values in the transcriptome database.

### qRT-PCR Validation

First-strand cDNA was synthesized from an equal amount of RNA using an EasyScript First-Strand cDNA Synthesis SuperMix (TransGen, Beijing, China). The qRT-PCR primers were designed for four selected genes encoding effector candidates with conserved motifs (Supplementary Table S1). The wheat leaf rust *PtActin* gene (GenBank accession OAV91054) was utilized as an internal reference gene. The qRT-PCR reactions were conducted using a TransStart^®^ Top Green qPCR SuperMix (TransGen, Beijing, China) with a Roche LightCycler96 qRT-PCR machine (Roche, Basel, Switzerland). Melting curves were generated by the machine to evaluate the specificity of the PCR products. The transcriptional abundances of genes encoding effector candidates were quantitated relative to that of the *PtActin* gene following the 2^–ΔCt^ method (Schmittgen and Livak, 2008).

### Gene Cloning and Yeast Two-Hybrid (Y2H) Assay

Using cDNA synthesized from wheat leaves of “Chinese Spring” inoculated with *Pt* pathotype PHTT(P) at 8 dpi, full length ORFs of 28 genes encoding 12 PNPi-like, 9 Y/F/WxC, and 7 CFEM effector candidates were cloned into the pENTR^TM^ TOPO^®^ vector (Invitrogen, Carlsbad, CA, United States). Genes encoding PNPi-like effector candidates were moved to Y2H vectors pLAW10 (binding domain, BD) and pLAW11 (activation domain, AD), respectively, as described (Yang et al., 2013). Potential signal peptides were truncated from the effector candidates to avoid the secretion of the protein from the Y2H system (Supplementary Table S1). Next, Y2H vectors carrying the full-length ORF of the *wNPR1* gene (wNPR1-BD and wNPR1-AD) described in our previous study (Wang et al., 2016) were co-transformed with the effector-recombined Y2H vector into yeast using the LiAc/SS carrier DNA/PEG method (Gietz, 2014). Co-transformants were initially selected on synthetic dropout (SD) selection media lacking leucine and tryptophan (SD–Leu–Trp), and then assayed on SD selection media lacking leucine, tryptophan, histidine, and adenine (SD–Leu–Trp–His–Ade).

### Transient Expression Assay

Full-length ORFs encoding 9 Y/F/WxC and 7 CFEM effector candidates were engineered into the PVX vector pGR107 (35S:Gene-GFP) as described (Zhao et al., 2018). The natural stop codons of the tested effector candidate genes were included (Supplementary Table S1). PVX vectors expressing the mouse cell death inducer BAX (Lacomme and Santa Cruz, 1999), *Phytophthora infestans* hypersensitive response elicitor INF1 (Kamoun et al., 1997), and GFP alone (empty pGR107) were derived from previous studies and employed as controls (Zhao et al., 2018). The recombinant vector was transformed into *Agrobacterium tumefaciens* strain EHA105 using the freeze/thaw method (Wise et al., 2006). The transformants were initially selected on LB medium with Kanamycin (50 μg/mL) and Rifampicin (50 μg/mL), and further validated by PCR test. Positive clones of *A. tumefaciens* transformants were inoculated in liquid LB medium and cultivated for 2 days. The transformed agrobacteria samples were collected by a brief centrifugation and suspended in 10 mM MgCl_2_ to OD_600_ = 0.2. Leaves from 4–6-week-old seedling plants of *Nicotiana benthamiana* were initially infiltrated with *A. tumefaciens* transformants carrying candidate effectors, and then infiltrated with BAX at the same site 24 h later. A cell death phenotype was observed around 3–5 days post-inoculation with BAX in the GFP alone control. For each of the effector candidates, this complete experiment was systemically repeated twice, for a total of three biological replicates. The cell death phenotype was recorded every 12 h.

To explore possible roles of the candidate effectors with conserved motifs during the early stage of plant defense response, tobacco leaves infiltrated with transformed *A. tumefaciens* were collected at 24 h-post inoculation (hpi) and stained using nitroblue tetrazolium (NBT) to visualize the accumulation of superoxide anion (O_2_^–^). The staining protocol was modified from a previous study (Wang et al., 2007). Briefly, tobacco leaves were collected and soaked in 10 mM NaN_3_ and 10 mM potassium phosphate buffer (pH 7.8) with 0.1% NBT (w/v) for 24 h. Next, samples were decolored in boiling 95% ethanol for 10 min. The percentage of stained area in each leaf was determined using ASSESS software (Lamari, 2008) and a Dunnett’s test was conducted using SAS software v9.4.

## Results

### *Pt* Pathotype PHTT(P) Was Designated Based on Its Virulent Profile

Urediospores of *Pt* were collected from wheat fields and then isolated in a greenhouse. A total of 20 wheat differential hosts carrying a single leaf rust resistance (*Lr*) gene were employed to clarify the virulent profile of the *Pt* isolate. Based on the observed leaf rust phenotypes (Supplementary Table S2) and the modified naming code for *Pt* pathotypes (Kolmer and Hughes, 2016), we temporally designated the *Pt* isolate as PHTT(P), which was reported as one of the predominant virulent pathotypes in China in both 2014 and 2015 (Zhang et al., 2020). Among the differential hosts screened, a few wheat isogenic lines carrying the *Lr2a*, *Lr9*, *Lr24*, and *Lr28* genes showed resistance to this *Pt* pathotype.

We then tested the phenotype of *Pt* pathotype PHTT(P) on the wheat cultivar “Chinese Spring,” which was recently used as the model of common wheat in a large-scale genome sequencing project (Appels et al., 2018). Like most of the differential hosts, wheat cultivar “Chinese Spring” was susceptible to *Pt* pathotype PHTT(P) as evidenced at 10 dpi.

### RNA-Seq Analysis Was Applied on *Pt* Pathotype PHTT(P) During Different Stages of Infection

To determine the virulent mechanism of *Pt* pathotype PHTT(P), RNA-seq analysis was performed on samples collected from infected leaves of the susceptible wheat cultivar “Chinese Spring” at 4, 6, and 8 dpi. Samples collected from the germinated urediospores (germ tube, GT) of *Pt* pathotype PHTT(P) were used as controls. Three biological replicates for each material were sampled and a total of 12 RNA samples were sent for 12-Gb transcriptome sequencing (Supplementary Table S3). Approximately 22–76 million 150-bp pair-end reads were obtained for each sample and mapped to the genome sequence of “*Pt* 1-1 BBBD Race 1” (Supplementary Table S4). For the transcriptome assembly, a total of 17,976 genes, including 2,284 “novel” transcripts, were annotated. The protein database of the “*Pt* 1-1 BBBD Race 1” genome and deduced proteins from these “novel” transcripts were combined into a single protein database that was used for further analyses. Biological replicates with significantly correlated (*R*^2^ > 0.92) gene expression levels (Supplementary Figure S3) were selected for further analysis. The transcriptional accumulation of genes was predicted based on the fragments per kilobase of transcript per million mapped reads (FPKM) value. Differentially expressed genes (DEGs) were identified by DESeq2. All raw data were uploaded to NCBI as BioProject PRJNA605036.

### DEGs of *Pt* Pathotype PHTT(P) During the Infection Process

**FIGURE 1:**
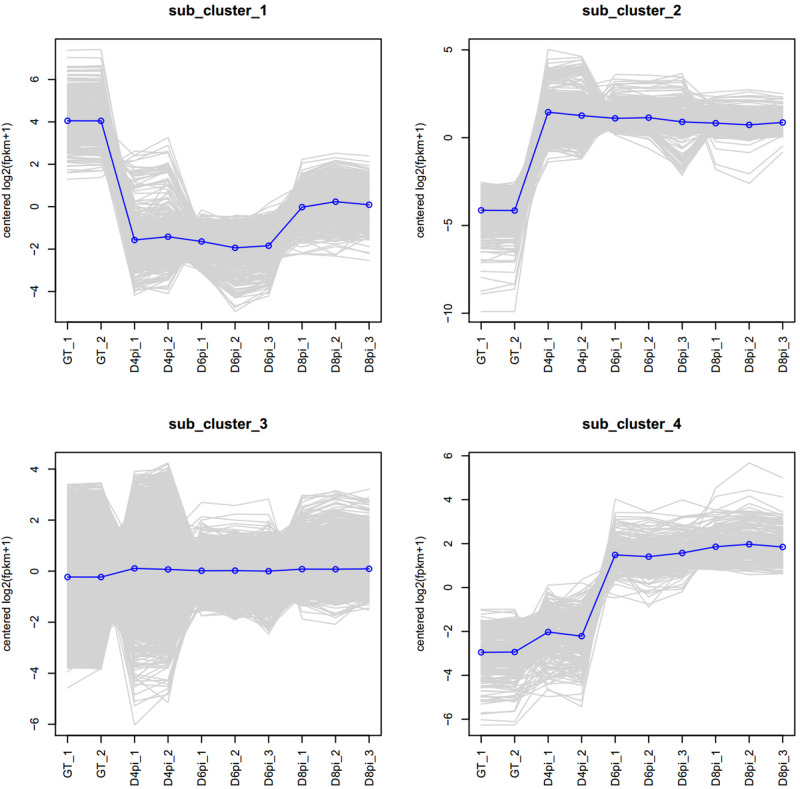
Clusters of *Pt* genes based on gene expression patterns. A total of 17,976 genes, including 2,284 “novel” transcripts, were annotated in the transcriptome. Four sub-clusters of genes were classified based on the observed expression patterns during rust infection. The sub-clusters are genes highly expressed in germ tubes (GT, sub_cluster_1), genes induced upon infection (sub_cluster_2), genes not induced (sub_cluster_3), and genes induced at late stage of rust infection (sub_cluster_4).

Based on the most commonly observed expression patterns, four sub-clusters of genes were classified (Figure 1): genes highly expressed in germ tube (GT, sub_cluster_1), genes induced upon infection (sub_cluster_2), genes not induced (sub_cluster_3), and genes induced at late stage of rust infection (sub_cluster_4). Significantly upregulated genes (Log2FoldChange > 1, adjust *p*-value < 0.05) in comparisons of “4 dpi vs. GT,” “6 dpi vs. GT,” and “8 dpi vs. GT” were combined as co-upregulated DEGs. A total of 3,149 non-redundant co-upregulated DEGs were identified, including genes encoding secreted proteins, sugar transporters, amino acid permeases, and protein kinases (Supplementary Table S5). On the other hand, a total of 1,613 non-redundant co-downregulated DEGs (Log2FoldChange < −1, adjust *p*-value < 0.05) were highly expressed in GT, including genes encoding hydrolases, protein kinases, and reverse transcriptases (Supplementary Table S6).

**FIGURE 2:**
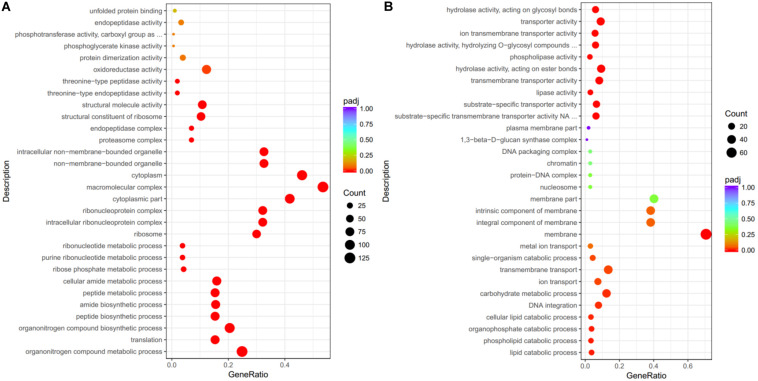
Gene ontology (GO) annotation for DEGs. The DEGs were categorized by their GO annotations and classified into three main categories: biological process, cellular component, and molecular function. **(A)** Significantly co-upregulated DEGs upon *Pt* infection were enriched in biological process of “oxidoreductase activity” and predicted to be localized in “macromolecular complex”. **(B)** For DEGs highly expressed in germ tubes of *Pt* uredospores, genes were annotated with “transporter” activity and predicted to be localized in “membrane.” The *x*-axis indicates the ratio in each category.

Although many DEGs were annotated as hypothetical proteins, the GO annotations of the co-upregulated DEGs revealed enrichment of genes with “oxidoreductase activity,” “structural molecule activity,” and “structural constituent of ribosome.” Proteins encoded by these co-upregulated DEGs were predicted to be localized in “macromolecular complex” and involved in “organonitrogen compound metabolic/biosynthetic process” (Figure 2A). For the co-downregulated DEGs, the encoded proteins were predicted to be localized in the “membrane,” and involved in “transmembrane transport” and “carbohydrate metabolic process” (Figure 2B).

SignalP was applied to identify secreted proteins encoded by the 3,149 co-upregulated DEGs. Secreted proteins were subjected to further evaluation of the effector probability using EffectorP (workflow presented in Supplementary Figure S1A). A total of 464 secreted proteins were encoded by the co-upregulated DEGs, with 79 of them also predicted as possible effectors by EffectorP (Supplementary Table S7).

### Genome-Wide Identification of RXLR-Like Effector Candidates From the Wheat Leaf Rust Fungus

**FIGURE 3:**
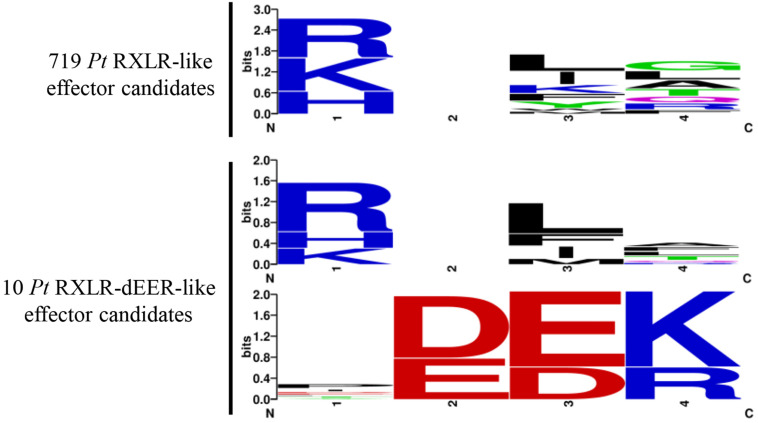
Hidden Markov models for the conserved regions of *Pt* RXLR-like effector candidates identified in the current study. A total of 719 RXLR-like effector candidates were identified from the wheat leaf rust fungus, including 10 proteins containing a complete RXLR-dEER-like motif. Hidden Markov models for the conserved regions of 719 RXLR-like and 10 RXLR-dEER-like effector candidates were generated using Weblogo.

We previously identified a virulent wheat stripe rust effector PNPi with a N-terminal RXLR-like (RSLL-DEEP) region and a C-terminal DPBB_1 conserved domain (PF03330) that suppressed plant systemic acquired resistance by directly targeting wheat NPR1 protein (Wang et al., 2016). To identify more RXLR-like effector candidates from the wheat leaf rust fungus, the combined protein database was analyzed using hmmsearch with the downloaded oomycete RXLR motif (PF16810) and Regex code of RXLR-like motif with all functional substitutions ([RKH]X[LIMFYWK][RALQGTF]) reported in a previous study (Kale et al., 2010). A total of 719 secreted proteins containing an N-terminal RXLR-like motif were identified, with 205 of these also predicted as possible effectors by EffectorP. There are 20 RXLR-like effector candidates contain an identical RXLR sequence (Supplementary Table S8). All the RXLR-like effector candidates were further screened using a Regex code of dEER-like motif ([ED][ED][KR]) derived from a previous research (Haas et al., 2009), which resulted in the discovery of 10 secreted proteins containing a complete RXLR-dEER-like motif (Supplementary Table S8). Hidden Markov models for the conserved regions of 719 RXLR-like and 10 RXLR-dEER-like effector candidates were visualized using Weblogo (Figure 3).

### Four PNPi-Like Effector Candidates Showed Protein Interaction With Wheat NPR1

**FIGURE 4:**
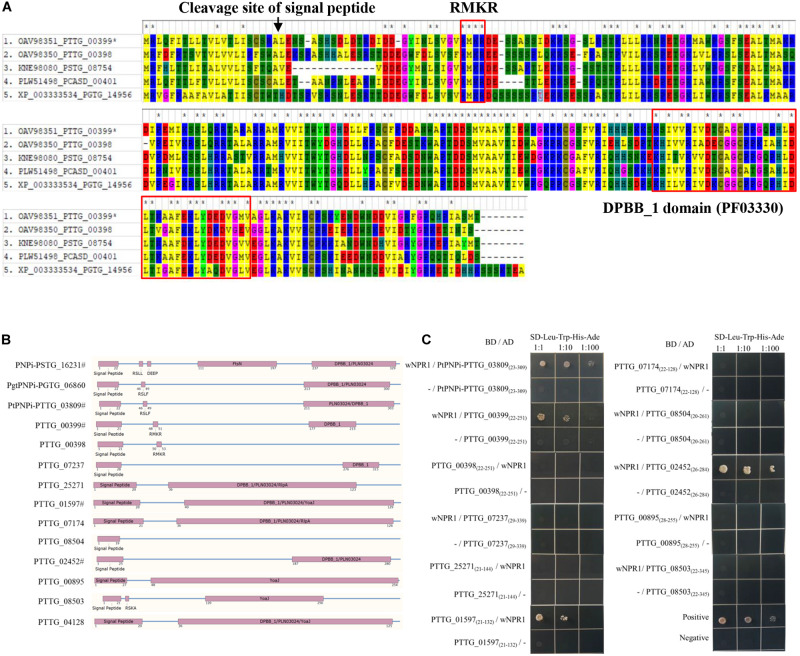
Identification of PNPi-like effector candidates from the wheat leaf rust fungus. **(A)** Sequence alignment of wheat leaf rust PNPi-like effector candidate PTTG_00399 and its closest homologs from related rust species. **(B)** The protein structure was annotated to indicate the signal peptide, RXLR-like region, and DPBB_1 conserved domain for each of the putative PNPi-like effectors. **(C)** Yeast transformants co-expressing different bait and prey constructs were assayed on SD–Leu–Trp–His–Ade.

We further noticed that one of the protein hits from oomycete RXLR hmmsearch, PTTG_00399 (*E*-value = 0.0085, Figure 4A), and the other five RXLR-like effector candidates identified by Regex have a C-terminal DPBB_1 conserved domain (PF03330). This domain in PNPi protein allows physical interaction with the C-terminal NPR1/NIM1-like domain (PF12313) of wheat NPR1 protein (Wang et al., 2016). We speculated that more PNPi-like effector candidates with DPBB_1 domain might co-target wheat NPR1 protein. Subsequent hmmsearch using DPBB_1 domain (PF03330) and homology analysis identified a total of 19 PNPi-like secreted proteins with similar structure (Table 1). Polygenetic analysis indicated that these PNPi-like effector candidates were conserved among different rust species (Supplementary Figure S4).

**TABLE 1:** Genome-wide prediction of PNPi-like effector candidates in the wheat leaf rust fungus.

				EffectorP	Effector	GT	4 dpi	6 dpi	8 dpi
Source	Gene accession	RXLR-like region	Conserved domain	prediction	probability	FPKM	FPKM	FPKM	FPKM
PNPi	PstPNPi_PSTG_16231*^#^	RSLL-DEEP	DPBB_1, FtsN/SPOR/DUF4770	Non-effector	0.975	N. A.	N. A.	N. A.	N. A.
	PgtPNPi_PGTG_17077	RSLF	DPBB_1/PLN03024	Non-effector	0.951	N. A.	N. A.	N. A.	N. A.
	PtPNPi_PTTG_03809*^#^	RSLF	DPBB_1	Non-effector	0.956	5.20	51.00	212.80	76.60
PNPi-like	PTTG_00399*^#^	RMKR	DPBB_1	Non-effector	0.509	2392.40	12.10	100.10	368.90
proteins	PTTG_00398*	RMKR	N. A.	Non-effector	0.611	326.90	158.20	15.10	27.20
	PTTG_06392	N. A.	DPBB_1	Non-effector	0.931	0.80	0.00	0.10	0.10
	PTTG_07237*	N. A.	DPBB_1	Non-effector	0.945	0.00	0.80	0.50	1.20
	PTTG_25271*	N. A.	DPBB_1/PLN03024/RlpA	Effector	0.592	5.90	8.10	43.10	65.60
	PTTG_04128*	N. A.	DPBB_1/PLN03024/YoaJ	Effector	0.645	0.10	0.10	2.80	31.10
	PTTG_01597*^#^	N. A.	DPBB_1/PLN03024/YoaJ	Effector	0.945	3.00	0.10	14.60	32.50
	PTTG_07174*	N. A.	DPBB_1/PLN03024/RlpA	Effector	0.626	10.20	0.20	100.00	276.90
	PTTG_08504*	N. A.	N. A.	Effector	0.691	0.10	19.10	56.30	16.70
	PTTG_02452*^#^	N. A.	DPBB_1/PLN03024	Non-effector	0.769	4.60	2.80	93.30	168.90
	PTTG_00895*	N. A.	YoaJ	Effector	0.599	0.00	0.00	0.30	0.90
	PTTG_08503*	RSKA	YoaJ	Non-effector	0.913	1.50	480.40	1389.00	156.20
	PTTG_04067	RRLT	DPBB_1/PLN03024	Non-effector	0.957	1409.90	7.40	5.10	50.10
	PTTG_00105	N. A.	DPBB_1/PLN03024/RlpA	Non-effector	0.695	0.90	0.10	0.40	0.20
	PTTG_00032	N. A.	DPBB_1/PLN03024/YoaJ/	Non-effector	0.598	0.40	0.70	0.50	0.60
	PTTG_06113	N. A.	PLN03024	Non-effector	0.918	328.30	146.90	36.60	46.60
	PTTG_02455	KGKL	DPBB_1	Effector	0.738	10.30	1.00	1.90	1.50
	PTTG_09546	N. A.	YoaJ	Effector	0.829	0.40	2.60	0.70	0.70
	PTTG_03028	N. A.	YoaJ	Effector	0.65	33.40	1.10	0.40	5.20

*Effector candidates selected for gene cloning and functional characterization. ^#^PNPi-like effector candidates that showed interaction with wheat NPR1 protein in the Y2H assay.

The expression patterns of genes encoding these PNPi-like effector candidates were profiled based on FPKM values, and 12 of them were significantly upregulated during the infection process of *Pt* pathotype PHTT(P) (Table 1), suggesting these genes may encode effectors. We then cloned these 12 genes encoding PNPi-like effector candidates (Figure 4B) and tested their potential interactions with wheat NPR1 protein by Y2H assay. The signal peptides of the effector candidates were truncated to avoid the secretion of the protein. All effector candidates were initially constructed into the pGADT7(AD) vector and then co-transformed with wNPR1-pGBKT7 (BD) into yeast. Several effector candidates exhibited self-activation of the AD vector, so these were subsequently cloned into the pGBKT7 vector and tested against wNPR1-pGADT7 in yeast. Four PNPi-like effector candidates showed interactions with wNPR1 under SD–Leu–Trp–His–Ade selection media (Figure 4C). We did not observe any growth defects of yeast co-transformants on non-interaction specific media (SD–Leu–Trp), except for PTTG_04128-pGBKT7 co-transformants, which did not grow.

### CRN-Like, Y/F/WxC, and CFEM Effector Candidates Were Identified From the *Pt* Pathotype PHTT(P)

**FIGURE 5:**
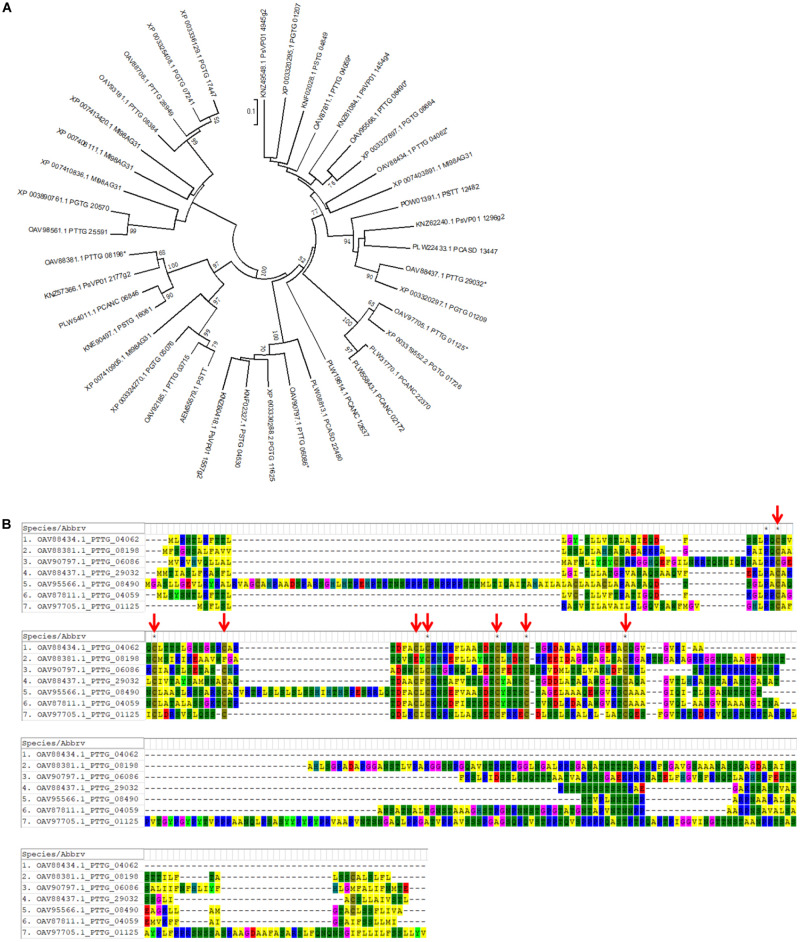
Identification of wheat leaf rust effector candidates with CFEM motif. Using HMM feature and homology analysis, nine secreted proteins with CFEM motif were identified from the wheat leaf rust fungus. **(A)** A phylogenetic tree of PtCFEM effector candidates and their closest homologs from related rust species was generated by MEGA software. Seven genes selected for further cloning and functional characterization are labeled with asterisks (*). **(B)** Protein sequences of the seven PtCFEM effector candidates were aligned using MUSCLE method. Conserved Cysteine residues are indicated with red arrows.

Effector candidates with other conserved motifs, including LFLAK (CRN), Y/F/WxC, CFEM, LysM, EAR, [SG]-P-C-[KR]-P, and ToxA, were explored following a similar workflow (Supplementary Figure S1B). Briefly, the hmm features for the conserved motifs of LysM (PF01476), EAR (PF07897), CFEM (PF05730), and ToxA (PF11584) were directly downloaded from the pfam website. Conserved regions from 141 oomycete CRN, 54 barley powdery mildew Y/F/WxC, and 30 *Fusarium* [SG]-P-C-[KR]-P effectors were collected from previous publications (Godfrey et al., 2010; Sperschneider et al., 2013; Stam et al., 2013) and employed to generate the hmm features using hmmbuild. Regex codes were generated for the conserved motifs of LFLAK (CRN), Y/F/WxC, and [SG]-P-C-[KR]-P based on the corresponding hidden Markov models (Supplementary Figure S2). Using hmmsearch, Regex, and homology analysis, we identified 19 CRN-like (Supplementary Table S9), 138 Y/F/WxC (Supplementary Table S10), and 9 CFEM (Table 2) effector candidates from the combined protein database. We did not detect any HVLVxxP-like motif in the 19 *Pt* CRN-like effector candidates. Phylogenetic analysis of PtCFEM with their closest homologs in other species indicated conservation of CFEM effectors among different rust species (Figure 5A). Additionally, at least eight conserved cysteine (C) residues were found in the sequence alignment (Figure 5B), which displayed a common feature of CFEM effectors. We did not find any secreted proteins containing conserved motifs of LysM, EAR, [SG]-P-C-[KR]-P, and ToxA.

**TABLE 2:** Genome-wide identification of secreted proteins with CFEM conserved motif from the wheat leaf rust fungus.

	Hmmsearch	EffectorP	Effector		GT	4 dpi	6 dpi	8 dpi
Gene accession	*E*-value	prediction	probability	Conserved motif (pfam)	FPKM	FPKM	FPKM	FPKM
PTTG_04062*	3.3e−13	Effector	0.846	CFEM domain (PF05730)	0.00	0.00	0.00	0.00
PTTG_06086*	1.3e−08	Effector	0.642	CFEM domain (PF05730)	0.50	0.14	13.12	23.18
PTTG_01125*	4.8e−08	Non-effector	0.989	CFEM domain (PF05730)	375.99	88.83	7.41	12.57
PTTG_08198*^+^	2.2e−06	Non-effector	0.919	CFEM domain (PF05730)	122.06	902.42	671.28	564.84
PTTG_29032*	1.1e−11	Non-effector	0.911	CFEM domain (PF05730)	4352.47	31.40	11.24	301.30
PTTG_08490*	3.4e−08	Non-effector	0.88	CFEM domain (PF05730)	72.95	2.12	32.48	50.17
PTTG_04059*	6.8e−14	Non-effector	0.795	CFEM domain (PF05730)	1158.27	539.55	275.46	205.49
PTTG_28949	8e−05	Non-effector	0.668	Glycosyl hydrolase family 61 (PF03443) CFEM domain (PF05730)	0.06	0.75	0.76	0.86
PTTG_03715	0.52	Non-effector	0.968	Fasciclin domain (PF02469) CFEM domain (PF05730, insignificant)	98.67	19.20	141.82	281.87

*Effector candidates selected for gene cloning and initial functional characterization. ^+^Effector candidates that promoted cell death triggered by BAX protein and accumulation of ROS in tobacco leaves.

### The Expression Profiles of Selected Effector Candidates Were Validated by qRT-PCR Assay

**FIGURE 6:**
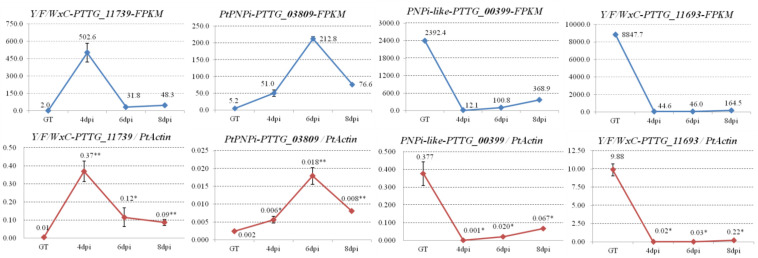
The expression patterns of four selected effector candidates during *Pt* infection were validated by qRT-PCR assay. Four genes encoding effector candidates with different expression patterns were selected. Their expression patterns were generated based on corresponding FPKM values from the transcriptome database. The transcript levels of the four selected effector candidates during *Pt* infection at 4, 6, and 8 dpi were determined by qRT-PCR assay. Samples collected from the germ tubes (GT) of *Pt* uredospores served as a control. The transcript levels for all genes were expressed as linearized fold-*PtActin* levels, which were calculated according to the formula 2^(ACTIN CT–TARGET CT)^. Data were expressed as mean values ± SE from four biological replicates. An asterisk (*P < 0.05, **P < 0.01) indicates a significant difference between the control and infection samples by Dunnett’s test.

Distinct samples than those used for the RNA sequencing were collected from germinated urediospores of *Pt* pathotype PHTT(P) and *Pt*-infected leaves of susceptible wheat cultivar “Chinese Spring” at 4, 6, and 8 dpi. *PtActin* (GenBank accession OAV91054) was employed as an internal reference gene to compare expression levels. The expression levels of genes in the germinated urediospores served as control. Four genes encoding effector candidates with different expression patterns were selected for qRT-PCR validation. The expression of *PtY/F/WxC_PTTG_11739* was significantly induced during early stage (4 dpi) of *Pt* infection, and there was high expression of *PtPNPi_PTTG_03809* in the late stage (6 dpi). *PNPi-like_PTTG_00399* and *PtY/F/WxC_PTTG_11693* showed higher expression in the germinated urediospores. These results were similar with the gene expression patterns predicted based on their FPKM values (Figure 6).

### CFEM Effector Candidate PTTG_08198 Accelerated the Progress of Cell Death and Promoted the Accumulation of ROS

**FIGURE 7:**
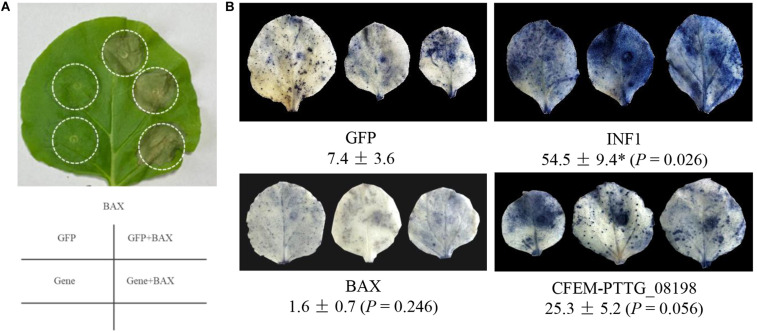
Functional characterization of leaf rust effector candidates with conserved motifs of Y/F/WxC and CFEM. **(A)** Sixteen effector candidates with conserved motifs (9 Y/F/WxC and 7 CFEM) were transiently expressed in *Nicotiana benthamiana* using *Agrobacterium* infiltration. An *Agrobacterium* transformant carrying *35S:BAX* was infiltrated 48 h after the initial infiltration of *35S:GFP* (negative control) and *35S:Effector*. None of the 16 tested effector candidates showed suppression of cell death triggered by the expression of *BAX* gene in tobacco leaves. Phenotype of BAX-induced cell death in tobacco leaves pre-expressing a CFEM effector candidate *PTTG_08198* was observed 24 h earlier than those expressing *35S:GFP* and other effector candidates. **(B)** Tobacco leaves were fully infiltrated with *Agrobacterium* transformant carrying *35S:GFP* (negative control), *35S:INF1* (positive control), *35S:BAX*, or *35S:CFEM-PTTG_08198*, respectively. Infiltrated leaves were collected at 24 hpi and subjected to NBT staining to visualize the accumulation of ROS. The percentage of the superoxide anion (O_2_^–^) accumulation area in the infiltrated leaf was calculated using the ASSESS software v2.0. The asterisk (*P < 0.05) indicates the significance of the differences between the GFP control and effector by Dunnett’s test.

The coding regions of 9 Y/F/WxC (Supplementary Table S10) and 7 CFEM (Figure 5 and Table 2) effector candidates were separately cloned into a plant expression vector (35S:Gene, T-DNA) and transiently expressed in *N. benthamiana* using *Agrobacterium* infiltration. None of the constructs directly induced cell death in tobacco leaves (Figure 7A). We used the mammalian *BAX* gene (GenBank accession NP_031553) to trigger plant effector-triggered immunity (ETI), and then assayed effects of expression of these 16 effector candidates on plant cell death. *Agrobacterium* transformants carrying *35S:BAX* were infiltrated 48 h after the initial infiltration of *35S:GFP* (negative control) and *35S:Effector*. None of the 16 tested effector candidates with conserved motifs suppressed cell death triggered by the expression of *BAX* gene in tobacco leaves (Figure 7A). However, BAX-induced cell death was observed 24 h earlier in tobacco leaves pre-expressing the CFEM effector candidate *PTTG_08198* compared to leaves expressing *35S:GFP* or other effector candidates.

To further investigate the role of CFEM-PTTG_08198 in plant pathogen-associated molecular pattern (PAMP)-triggered immunity (PTI), tobacco leaves were fully infiltrated with *Agrobacterium* transformants carrying *35S:GFP* (negative control), *35S:INF1* (positive control), *35S:BAX*, or *35S:CFEM-PTTG_08198*. To visualize the accumulation of ROS, infiltrated leaves were collected at 24 hpi and subjected to NBT staining. Compared with the GFP control, transient expression of *INF1* resulted in significant higher accumulation of superoxide anion (O_2_^–^), whereas leaves expressing BAX protein showed rapid removal of ROS (Figure 7B). Relatively higher (*P* = 0.056) accumulation of ROS was observed in tobacco leaves expressing *CFEM-PTTG_08198* (Figure 7B).

## Discussion

Wheat leaf rust, caused by *P. triticina* (*Pt*), is one of the most severe and globally widespread fungal diseases in wheat (Huerta-Espino et al., 2011; Helfer, 2014). The *Pt* isolate PHTT(P) identified and sequenced here represents the major epidemic virulent pathotype in China in recent years (Zhang et al., 2020). Most of the designated wheat leaf rust resistance (*Lr*) genes have been compromised by this virulent rust pathotype (Supplementary Table S2), indicating that PHTT(P) may be a suitable model to study the virulence mechanism by which leaf rust suppresses various plant defenses.

Approximately 22–76 million reads were obtained from each sample in the current study (Supplementary Table S3), which was significantly more than previous RNA-seq assays (23–33 million reads) of wheat leaves infected with six different *Pt* pathotypes (Bruce et al., 2014). This first set of sequencing data from purified germ tubes of the wheat leaf rust fungus should be a valuable resource to understand the germination process and very early infection stage of this devastating pathogen. We identified 17,976 genes, including 2,284 “novel” transcripts, from our transcriptome assembly based on the reference genome of “*Pt* 1-1 BBBD Race 1” (Kiran et al., 2016). Testing multiple time points after infection, the expression patterns of *Pt* genes involved in the germination process (GT), early infection (4 and 6 dpi), and sporulation process (8 dpi) of the wheat leaf rust fungus were profiled on the basis of their FPKM values (Figure 1). The identified 3,149 co-upregulated DEGs associated with the *Pt* infection process were enriched for genes encoding secreted proteins, sugar transporters, amino acid permeases, and protein kinases (Supplementary Table S5). According to GO annotation, many of these genes were associated with “oxidoreductase activity” and the encoded proteins were predicted to be localized in “macromolecular complex” (Figure 2A). A total of 464 secreted proteins were encoded by these co-upregulated DEGs, with 79 of them also predicted as possible effectors by EffectorP (Supplementary Table S7). Previous studies identified 660 and 532 secreted proteins from the genome and transcriptome, respectively, of the wheat leaf rust fungus (Bruce et al., 2014; Kiran et al., 2016). Hundreds of genes encoding secreted proteins that are highly induced during infection have been identified from different rust species (Figueroa et al., 2016; Lorrain et al., 2019). Compared with related studies, effector candidates encoded by the co-upregulated DEGs identified here should be more directly associated with the infection process of the wheat leaf rust fungus.

Fungal or oomycete effectors with conserved motifs, such as RXLR, Y/F/WxC, CFEM, LysM, EAR, LFLAK-HVLVxxP (CRN), [SG]-P-C-[KR]-P, and ToxA, are considered “core effectors” that play crucial roles during pathogen infection (Liu et al., 2019). Many effectors with the RXLR motif from *P. sojae* function through a plant cell death-related pathway and contribute to pathogen virulence (Wang et al., 2011). Through systematic mutagenesis of the Avr1b RXLR motif, a broadened “RXLR-like” motif was defined and found in many intracellular fungal effectors such as *Melampsora lini* AvrL567 (RFYR), *Fusarium oxysporum* Avr2 (RIYER), *Leptosphaeria maculans* AvrLm6 (RYWT), and *Laccaria bicolor* MiSSP7 (RALG) (Kale et al., 2010; Rafiqi et al., 2010; Plett et al., 2011). The genome-wide identification of RXLR-like effector candidates from the wheat leaf rust fungus may provide further insight into the virulent mechanism of this destructive pathogen. An earlier study identified a wheat stripe rust effector candidate Ps87 containing a RXLR-like motif (KRLTG), which was found capable of delivering oomycete effector Avr1b into soybean leaf cells and carrying GFP into soybean root cells (Gu et al., 2011). In a previous study, we discovered a virulent rust effector, PNPi, with a RXLR-like motif (RSLL–DEEP) and a DPBB_1 conserved domain that suppressed systemic acquired resistance by directly targeting the wheat NPR1 protein (Wang et al., 2016). In this study of the wheat leaf rust fungus, we initially identified 719 RXLR-like effector candidates (Figure 3), including 20 secreted proteins containing an identical sequence of RXLR and 10 secreted proteins containing a complete RXLR-dEER-like motif (Supplementary Table S8).

We noticed additional RXLR-like effector candidates with a DPBB_1 conserved domain in their C-terminal regions. Subsequent hmmsearch using DPBB_1 conserved domain (PF03330) and homology analysis identified a total of 19 PNPi-like secreted proteins with similar structures (Table 1). Twelve of these PNPi-like effectors were selected for further characterization (Figure 4B), and four of them exhibited protein interaction with wheat NPR1 in Y2H assay (Figure 4C). NPR1 protein was predicted to be potentially targeted by various effectors, especially YopJ effectors with desumoylation activity (Sun et al., 2018). A bacterial type III effector AvrPtoB was reported to interact with and ubiquitinate NPR1 in *Arabidopsis* (Chen et al., 2017). Another RXLR effector, RXLR48 from *Phytophthora capsica*, associated with NPR1 and suppressed plant immune response (Li et al., 2019). The four wNPR1-interacting PNPi-like effector candidates from the wheat leaf rust fungus could be used as novel targets for both mechanism research and fungicide design.

A total of 19 CRN-like (Supplementary Table S9), 138 Y/F/WxC (Supplementary Table S10), and 9 CFEM (Figure 5 and Table 2) effector candidates were identified from the combined protein database. Oomycete pathogens produce abundant CRN effectors to manipulate plant immune responses and promote infection. One *P. sojae* CRN effector, PsCRN108, reprogrammed the expression of plant *HSP* genes by targeting their promoters (Song et al., 2016). Recent studies have also discovered a large number of CRN-like effector candidates in different fungal pathogens (Raffaele et al., 2010; Voß et al., 2018). A CRN-like effector RiCRN1from *Rhizophagus irregularis* acts in arbuscule development (Voß et al., 2018). The identified 19 CRN-like effector candidates may provide insight into the mechanism of virulence of the wheat leaf rust fungus.

The Y/F/WxC motif is present in the N-terminal region of various powdery mildew fungal effector candidates, positioned after the cleavage site of the signal peptide (Godfrey et al., 2010; Vela-Corcia et al., 2016). Barley powdery mildew Y/F/WxC effector candidates CSEP0081 and CSEP0254 were found to contribute to pathogen virulence (Ahmed et al., 2016). However, in initial tests of the nine selected Y/F/WxC effector candidates, we did not find any promotion or suppression of plant cell death (Figure 7A). Thus, the role of these effector candidates in the virulence of the wheat leaf rust fungus requires further exploration.

Fungal-specific CFEM effectors normally contain at least eight conserved cysteine residues (Figure 5B) and are considered extracellular avirulent effectors that triggering plant immunity responses (Catanzariti et al., 2006; Zhang et al., 2015). For example, a CFEM domain-containing protein BcCFEM1 from *Botrytis cinerea* directly induced chlorosis in *N. benthamiana* (Zhu et al., 2017). A CFEM effector found here, PTTG_08198, accelerated plant cell death triggered by BAX protein and induced higher accumulation of ROS in tobacco leaves (Figure 7B). Further investigations on the molecular receptor of this *Pt* CFEM effector should greatly improve our understanding of the avirulent mechanism of the wheat leaf rust fungus.

## Conclusion

We have initially profiled genes associated with the infection process of the *Pt* pathotype PHTT(P). Effector candidates with conserved motifs and other DEGs induced upon *Pt* infection identified in this study will be valuable resources to determine the molecular mechanisms of the wheat leaf rust fungus.

## Data Availability Statement

The datasets supporting the results of this article can be found in the article and its additional files. Raw sequence reads have been deposited in the NCBI Sequence Read Archive under the BioProject PRJNA605036.

## Author Contributions

XDW and XJW designed the experiments. SZ conducted most of the experiments. XS worked on the rust inoculation. WB helped with the gene cloning and vector construction. XDW generated the first and last draft of the manuscript. XY, DL, ZK, XJW, and XDW contributed to the revision of the manuscript. All authors read and approved the manuscript.

## Conflict of Interest

The authors declare that the research was conducted in the absence of any commercial or financial relationships that could be construed as a potential conflict of interest.

## Footnotes

1 http://www.cbs.dtu.dk/services/SignalP/

2 http://effectorp.csiro.au

3 http://pfam.xfam.org

4 http://weblogo.berkeley.edu/logo.cgi
