# Supplementary material for: Genome-Wide Identification of Effector Candidates With Conserved Motifs From the Wheat Leaf Rust Fungus Puccinia triticina
Source: Front Microbiol. 2020 Jun 3;11:1188. doi: 10.3389/fmicb.2020.01188 (PMC7283542; doi:10.3389/fmicb.2020.01188)
Supplement: Supplementary file 1 [file Data_Sheet_1.PDF]

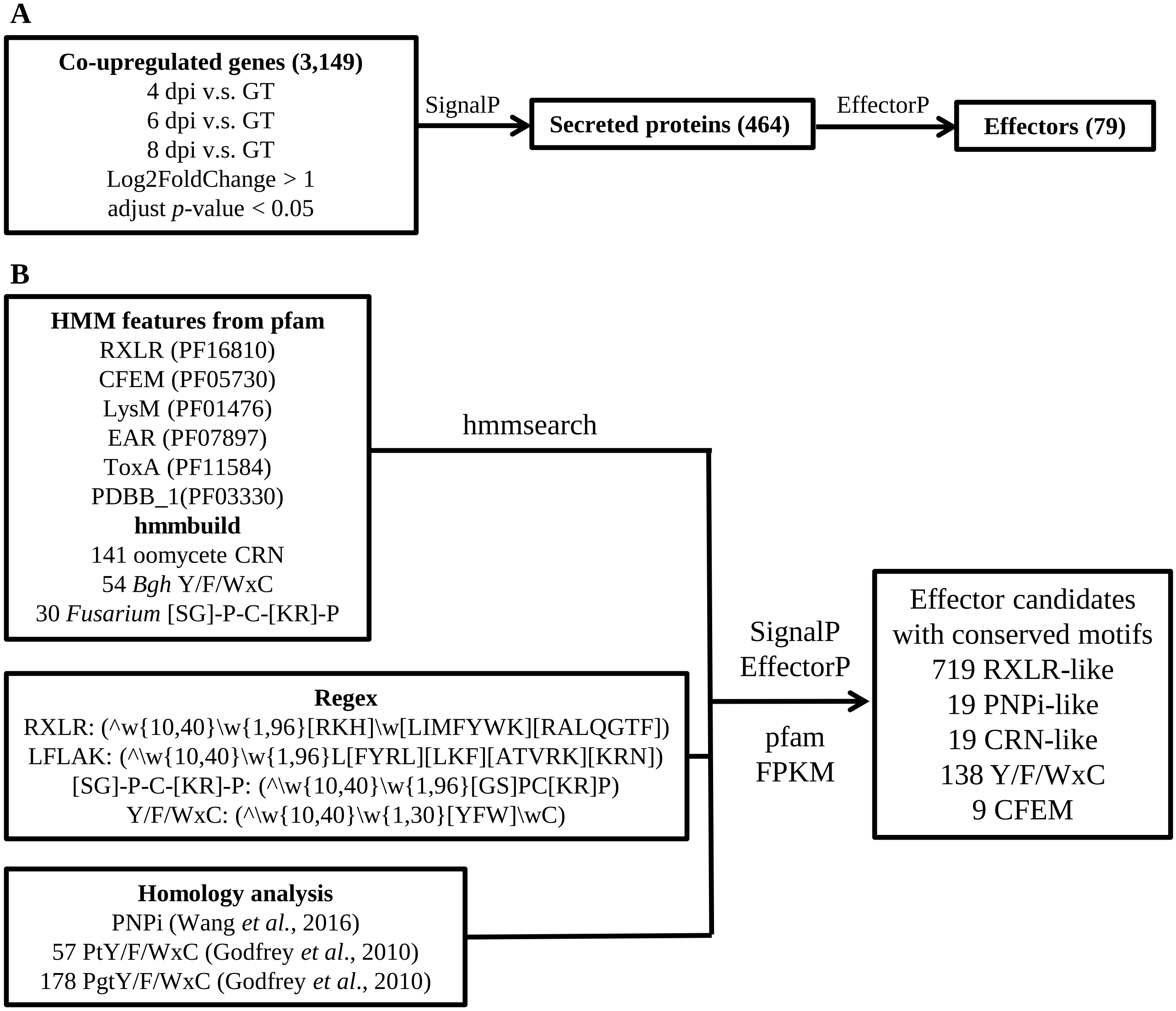

SUPPLEMENTARY FIGURE S1 | Workflow to identify effector candidates. (**A**) A total of 3,149 genes showed significant inductions upon *Pt* infection. The encoded proteins were screened using SignalP and 464 secreted proteins were annotated. All these secreted proteins were subjected to further evaluation of the effector probability using EffectorP. A total of 79 effector candidates encoded by those genes significantly induced during *Pt* infection were identified. (**B**) HMM features of RXLR (PF16810), LysM (PF01476), EAR (PF07897), CFEM (PF05730), ToxA (PF11584), PDBB_1 (PF03330) were directly downloaded from the pfam website (http://pfam.xfam.org). Whereas conserved regions of 141 oomycete CRN, 54 barley powdery mildew Y/F/WxC, and 30 *Fusarium* [SG]-P-C-[KR]-P effectors from previous publications, respectively, were utilized to generate the corresponding HMM features using hmmbuild. The combined protein database of the "*Pt* 1-1 BBBD Race 1" genome and deduced proteins from the "novel" transcripts of the presented transcriptome was initially screened using hmmsearch to identify proteins with any of the conserved motifs. Regex using presented code was conducted to identify more proteins with conserved motifs of RXLR, LFLAK (CRN), [SG]-P-C-[KR]-P, and Y/F/WxC. Homology analyses were performed using several previously reported rust effectors with consevered motif (PNPi, PtY/F/WxC, and PgtY/F/WxC) by local Blastp. SignalP was employed to detect the signal peptide of the protein. EffectorP was applied to evaluate the effector probability for each of the secreted protein. Conserved domains in each of the identified effector candidates were predicted using pfam. The expression patterns of all the effector candidates were profiled based on their FPKM values in the transcriptome database.

141 oomycete CRN

54 barley powdery mildew Y/F/WxC

30 *Fusarium* [SG]-P-C-[KR]-P

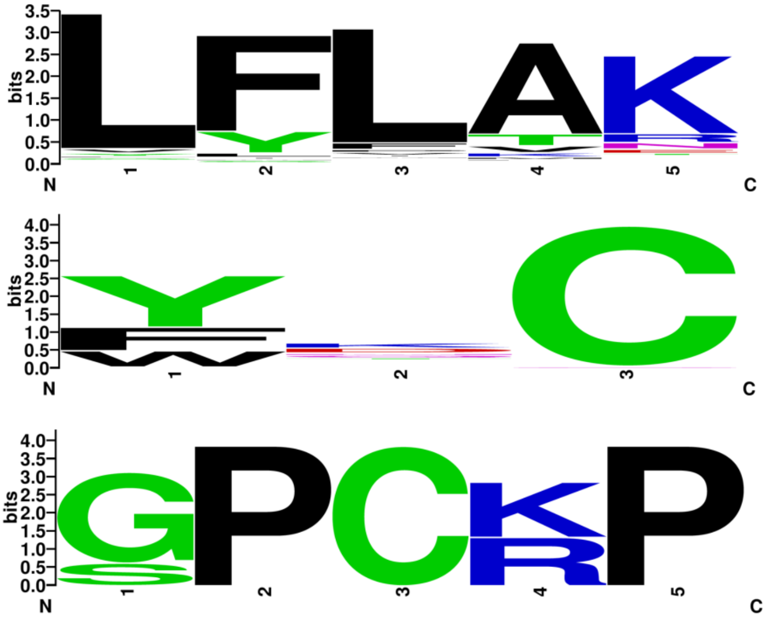

SUPPLEMENTARY FIGURE S2 | Conserved regions of 141 oomycete CRN, 54 barley powdery mildew Y/F/WxC, and 30 *Fusarium* [SG]-P-C-[KR]-P effectors utilized in hmmbuild and generation of Regex code. The hidden Markov models for these conserved motifs were visualized using Weblogo.

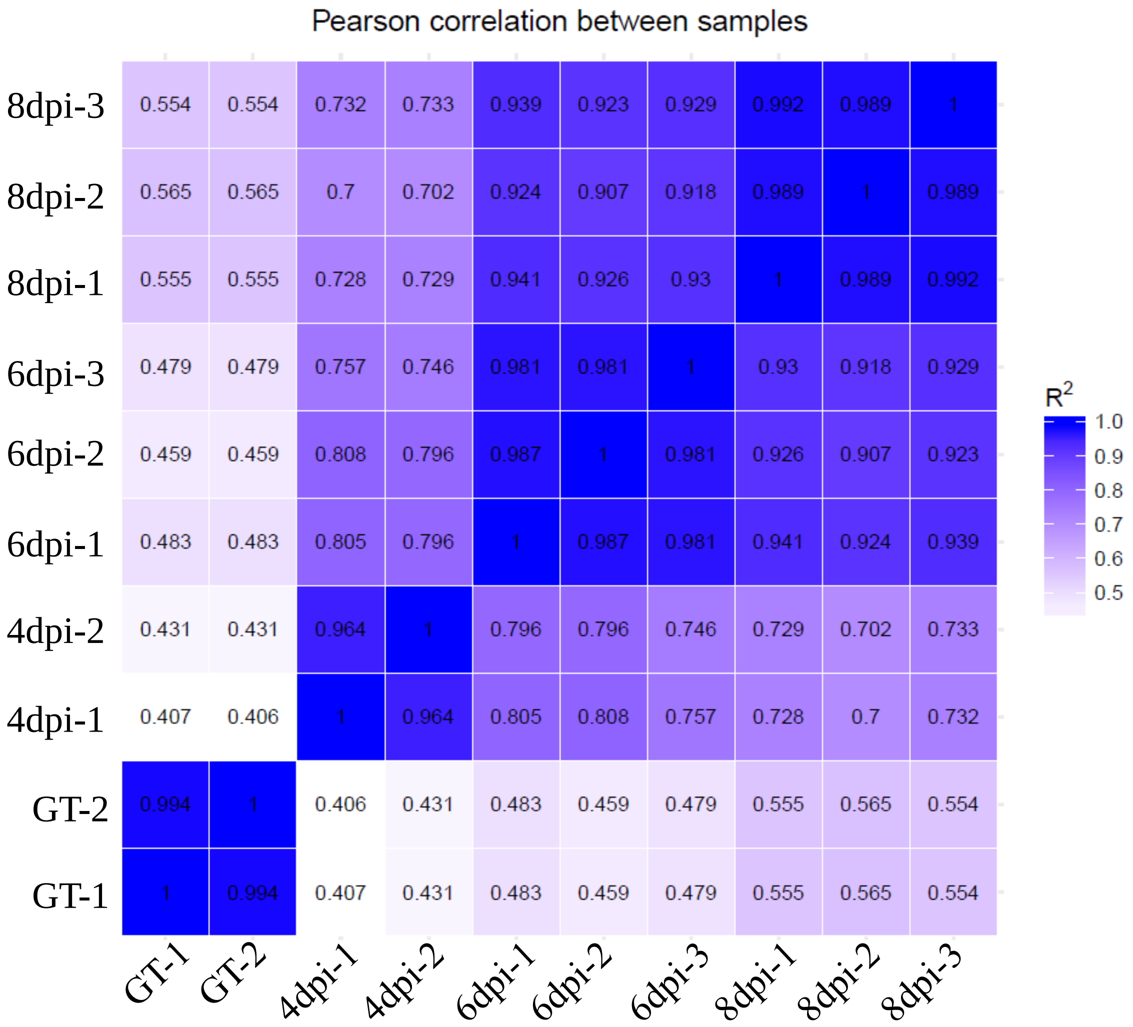

SUPPLEMENTARY FIGURE S3 | Pearson's correlations of the overall gene expression levels between biological replicates in the RNA-seq assay. Transcriptome analysis was applied on RNA samples from the germinated uredospores (GT) of *Pt* pathotype PHTT(P) and infected leaves of susceptible wheat cultivar "Chinese Spring" at 4, 6, 8 dpi. A number of 2-3 biological replicates for each of the materials were sent for 12-Gb RNA sequencing. Clear correlations of the overall gene expression levels between biological replicates were detected (R^2 > 0.92). GT: germ tube of *Pt* uredospores, dpi: days post-inoculation.

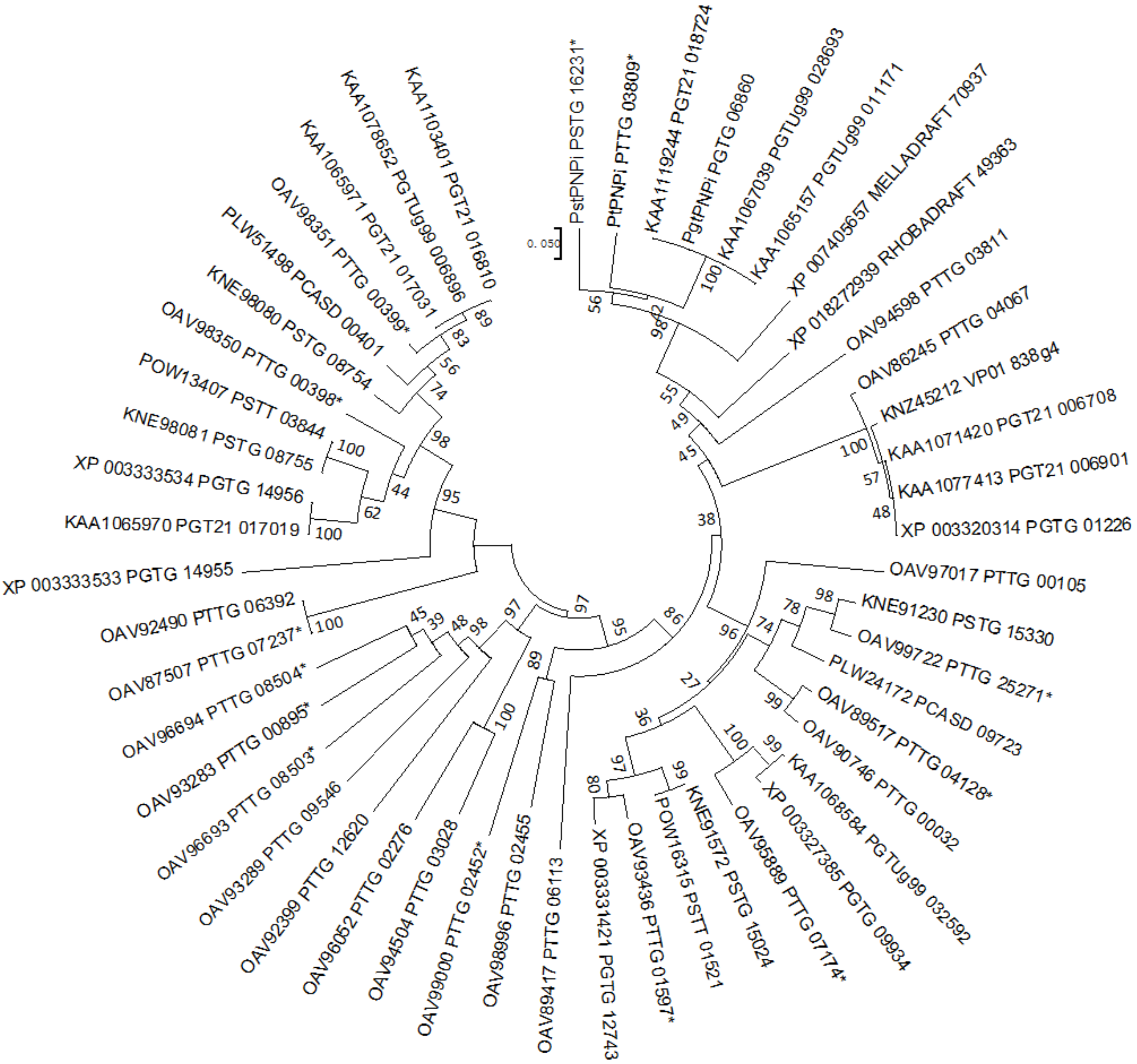

SUPPLEMENTARY FIGURE S4 | The phylogenetic tree of wheat leaf rust PNPi-like effector candidates. A neighbor-joining tree was constructed by MEGA software using protein sequences of wheat leaf rust PNPi-like effector candidates and their closest homologs from relative species. A total of twelve genes selected for further cloning and functional characterization are labeled with asterisks (*).
