# Supplementary material for: Genome-Wide Identification of Effector Candidates With Conserved Motifs From the Wheat Leaf Rust Fungus Puccinia triticina
Source: Front Microbiol. 2020 Jun 3;11:1188. doi: 10.3389/fmicb.2020.01188 (PMC7283542; doi:10.3389/fmicb.2020.01188)
Supplement: Supplementary file 2 [file Data_Sheet_2.pdf]

SUPPLEMENTARY TABLE S1 | Primers used in this study.

Primer usage	Primer Name	Primer sequences 5' to 3'	PCR product	GenBank accession
Transient expression assay	Y/F/WxC-PTTG_11693 -ORF-F	caccATGAATGCCGCTCCCTATG	369 bp	OAV87608
	Y/F/WxC-PTTG_11693 -ORF-R	TTATTTTGCCCAGCTGACTTTAT		
	Y/F/WxC-PTTG_25643 -ORF-F	caccATGTTTTCTCCATCATTAGTCCG	357 bp	OAV98383
	Y/F/WxC-PTTG_25643 -ORF-R	TCAGCCGGGACTATTTGGTT		
	Y/F/WxC-PTTG_12170 -ORF-F	caccATGGACTTCTGGATGTCGATCA	363 bp	OAV93362
	Y/F/WxC-PTTG_12170 -ORF-R	CTATGGGTAATGAGTCTTGGCTG		
	Y/F/WxC-PTTG_11739 -ORF-F	caccATGCTCTCCGTGAAAATTTTCTT	441 bp	OAV94198
	Y/F/WxC-PTTG_11739 -ORF-R	ATAGGCCAGCTTGCGTGATT		
	Y/F/WxC-PTTG_12171-ORF-F	caccATGAACTTTTCGACATCAATGAAT	357 bp	OAV93363
	Y/F/WxC-PTTG_12171-ORF-R	CTATGGGTAATGAGTCTTTGCGG		
	Y/F/WxC PTTG_30134 -ORF-F	caccATGCAATTCTCTAGTTTTTTGATCC	375 bp	OAV86017
	Y/F/WxC PTTG_30134 -ORF-R	TCAACGGCGGGGATTTG		
	Y/F/WxC-PTTG_28678 -ORF-F	caccATGCATCATTTCAAGCTTTGGG	372 bp	OAV89452
	Y/F/WxC-PTTG_28678-ORF-R	TCAGCGCGGGCGTGC		
	Y/F/WxC-PTTG_28615 -ORF-F	caccATGCATCTCGCGAAGCTTT	384 bp	OAV89701
	Y/F/WxC-PTTG_28615 -ORF-R	TCATCGGGATTTTTTCTTTCC		
	Y/F/WxC-PTTG_26924-ORF-F	caccATGCAGTCCTTCGCCCTTCT	387 bp	OAV94540
	Y/F/WxC-PTTG_26924-ORF-R	TCAGTTGTGATGTGATGGGGC		
	CFEM -PTTG_04059 -ORF-F	caccATGCTTTCCTACTCCAACACCTT	582 bp	OAV87811
	CFEM -PTTG_04059-ORF-R	TCAGATCATCAAGAGCGAGGAA		
	CFEM -PTTG_04062-ORF-F	caccATGCTTCCCAATACGCTGC	306 bp	OAV88434
	CFEM -PTTG_04062-ORF-R	TCATGCGGCAATCTTGACAC		
	CFEM -PTTG_29032-ORF-F	caccATGATGACCATCGCCAGCC	492bp	OAV88437
	CFEM -PTTG_29032-ORF-R	TTACAGGGTTGAGACAATTGCC		
	CFEM -PTTG_06086-ORF-F	caccATGGTGCCCGTGCATGTG	630	OAV90797
	CFEM -PTTG_06086-ORF-R	TTACTCGGTCATGTTGAAAATCAAA		
	CFEM -PTTG_08490-ORF-F	caccATGGGGGCGAGCCTTCTT	726 bp	OAV95566
	CFEM -PTTG_08490-ORF-R	TCAAGCGACGATCAAAAAGGA		
	CFEM -PTTG_01125-ORF-F	caccATGAGCTTCCTAAGTTTGCG	834 bp	OAV97705
	CFEM -PTTG_01125-ORF-R	CTAGACATACAAGAGGGATGAAAAT		
	CFEM -PTTG_08198-ORF-F	caccATGTTCTCCGGAAACTCTGCC	675 bp	OAV88381
	CFEM -PTTG_08198-ORF-R	CTAGAGGAAGAGGGAGAGTGCG		
Y2H assay	PNPi-like-PTTG_00399-EcoRI-AD-F	GAATTCCTTGAAAATTCGGCTAGTCATTC	690 bp	OAV98351
	PNPi-like-PTTG_00399-XhoI-AD-R	CTCGAGGGTCATCGAGGCAATGGG		
	PNPi-like-PTTG_07237-EcoRI-BD-F	GAATTCTCGGGCAAAACTTCAGG	933 bp	OAV87507
	PNPi-like-PTTG_07237-Pst1-BD-R	CTGCAG GAGTATCCGAGGACCATAGAG		
	PNPi-like-PTTG_25271-EcoRI-BD-F	GAATTCTCGCCCGTCTCCGACAA	372 bp	OAV99722
	PNPi-like-PTTG_25271-Pst1-BD-R	CTGCAG AACGAAGTACGCGATGCAGAT		
	PNPi-like-PTTG_04128-EcoRI-BD-F	GAATTCACCCCCCTATCTACTTGGAAAT	324 bp	OAV89517

	PNPi-like-PTTG_04128-Pst1-BD-R	CTGCAG TTGAAACTGCCACGAAATCG		
	PNPi-like-PTTG_01597-EcoRI-BD-F	GAATTCAGCGTTTCAAGGCCTTCC	336 bp	OAV93436
	PNPi-like-PTTG_01597-Pst1-BD-R	CTGCAG AGCCCAATGCCACGAAA		
	PNPi-like-PTTG_07174-EcoRI-BD-F	GAATTCCTTCCGCTACCCTCTACATCC	324 bp	OAV95889
	PNPi-like-PTTG_07174-Pst1-BD-R	CTGCAG CGTCAAATCCCAAGTGATCG		
	PNPi-like-PTTG_08504-Nde1-AD-F	CATATGAAGCCACTCAACTTGAGGC	726 bp	OAV96694
	PNPi-like-PTTG_08504-BamH1-AD-R	GGATCCGCAGTTCCCGACCGC		
	PNPi-like-PTTG_02452-Nde1-AD-F	CATATGGCAGACCTCCAAAACTTCATC	777 bp	OAV99001
	PNPi-like-PTTG_02452-BamH1-AD-R	GGATCCCATGCAGACGACCACGCT		
	PNPi-like-PTTG_00895-Nde1-BD-F	CATATGATCAGCTCTGGTGAGGTACATCATCATC	684 bp	OAV93283
	PNPi-like-PTTG_00895-Pst1-BD-R	CTGCAG ACAGTTTGCGGATCCCGGCTT		
	PNPi-like-PTTG_08503-EcoRI-AD-F	GAATTCTATTGGCCGCCTTTGAGAAGTAAAG	972 bp	OAV96693
	PNPi-like-PTTG_08503-BamH1-AD-R	GGATCCAGAACAGTTTCCGGAGGCCG		
	PNPi-like-PTTG_00398-Nde1-BD-F	CATATGCTCGAAAAAAGCCCTGCAAG	690 bp	OAV98350
	PNPi-like-PTTG_00398-BamH1-BD-R	GGATCCCGAGATGTTAATGGTTTCCTTCG		
qRT-PCR assay	PtPNPi_PTTG_03809-qRT-F	GAATGGTCCAAGCCAGAA	341 bp	OAV94594
	PtPNPi_PTTG_03809-qRT-R	TCATCAGCGGCAATACAA		
	PNPi-like-PTTG_00399-qRT-F	CGTCCATAGATAAACGCTCCG	321 bp	OAV98351
	PNPi-like-PTTG_00399-qRT-R	CGCACTTGGGCTTTCCTG		
	PtActin-qRT-F	ATCCACGAGACCACCTACAACT	219 bp	OAV91054
	PtActin-qRT-R	CAAGATAGAACCACCAATCCATAC		
	Y/F/WxC-PTTG_11693-qRT-F	AATGAAATGTTCGACCTATGCT	250 bp	OAV87608
	Y/F/WxC-PTTG_11693-qRT-R	GCCGTATCCAGTTGTAGTGCC		
	Y/F/WxC-PTTG_11739-qRT-F	TTTGACGGCTCTTGTCTTGC	180 bp	OAV94198
	Y/F/WxC-PTTG_11739-qRT-R	ACTCGATGTCCGGGTTGTTT		

SUPPLEMENTARY TABLE S2 | Virulent profile of *Pt* pathotype PHTT(P).

**Wheat isogenic line**	**Leaf rust phenotype**	**Designation**
Lr1	H	P
Lr2a	L	
Lr2c	H	
Lr3	H	
Lr9	L	H
Lr16	H	
Lr24	L	
Lr26	H	
Lr3ka	H	T
Lr11	H	
Lr17	H	
Lr30	H	
LrB	H	T
Lr10	H	
Lr14a	H	
Lr18	H	
Lr21	H	(P)
Lr28	L	
Lr39	H	
Lr42	H	

SUPPLEMENTARY TABLE S3 | Sequencing information for the transcriptome.

Sample	Raw reads	Clean reads	Clean bases	error_rate	Q20	Q30	GC_pct
GT_1	62641700	61621376	9.24G	0.03	97.25	92.85	55.04
GT_2	60274364	59106856	8.87G	0.03	97.18	92.71	55.02
GT_3	51066024	50015870	7.5G	0.03	96.95	92.24	54.87
4_dpi_1	24658448	23139562	3.47G	0.03	94.25	88.33	56.98
4_dpi_2	21585930	19689974	2.95G	0.04	93.55	87.45	58.07
4_dpi_3	38868136	37347224	5.6G	0.03	94.89	88.56	56.45
6_dpi_1	50796794	49492326	7.42G	0.03	96.43	91.5	54.82
6_dpi_2	58159750	56625754	8.49G	0.03	96.51	91.65	54.78
6_dpi_3	75846454	73981986	11.1G	0.03	96.69	91.91	54.38
8_dpi_1	66356108	64430044	9.66G	0.03	96.42	91.44	54.77
8_dpi_2	60202242	58860648	8.83G	0.03	96.79	92.16	54.09
8_dpi_3	69269956	67623462	10.14G	0.03	96.76	92.1	54.71

SUPPLEMENTARY TABLE S4 | Details of the transcriptome assembly.

sample	total_reads	total_map	unique_map	multi_map	read1_map	read2_map	positive_map	negative_map	splice_map	unsplice_map	proper_map
GT_1	61621376	51069014(82.88%)	49959493(81.07%)	1109521(1.8%)	25055324(40.66%)	24904169(40.41%)	24931811(40.46%)	25027682(40.62%)	21789849(35.36%)	28169644(45.71%)	46601186(75.63%)
GT_2	59106856	49202742(83.24%)	48081153(81.35%)	1121589(1.9%)	24142594(40.85%)	23938559(40.5%)	23991045(40.59%)	24090108(40.76%)	21058342(35.63%)	27022811(45.72%)	44895330(75.96%)
GT_3	50015870	43306979(86.59%)	42343215(84.66%)	963764(1.93%)	21295047(42.58%)	21048168(42.08%)	21144380(42.28%)	21198835(42.38%)	18254149(36.5%)	24089066(48.16%)	39676962(79.33%)
4_dpi_1	23139562	13421229(58.0%)	13238697(57.21%)	182532(0.79%)	6658377(28.77%)	6580320(28.44%)	6622925(28.62%)	6615772(28.59%)	5744896(24.83%)	7493801(32.39%)	12500508(54.02%)
4_dpi_2	19689974	9042244(45.92%)	8909336(45.25%)	132908(0.68%)	4468726(22.7%)	4440610(22.55%)	4456350(22.63%)	4452986(22.62%)	3849680(19.55%)	5059656(25.7%)	8418192(42.75%)
4_dpi_3	37347224	27230105(72.91%)	26804334(71.77%)	425771(1.14%)	13509914(36.17%)	13294420(35.6%)	13410316(35.91%)	13394018(35.86%)	11615406(31.1%)	15188928(40.67%)	25176834(67.41%)
6_dpi_1	49492326	39651152(80.12%)	39287181(79.38%)	363971(0.74%)	19734600(39.87%)	19552581(39.51%)	19635819(39.67%)	19651362(39.71%)	16029244(32.39%)	23257937(46.99%)	36929938(74.62%)
6_dpi_2	56625754	45022569(79.51%)	44627317(78.81%)	395252(0.7%)	22416094(39.59%)	22211223(39.22%)	22303327(39.39%)	22323990(39.42%)	18126739(32.01%)	26500578(46.8%)	42005586(74.18%)
6_dpi_3	73981986	59501095(80.43%)	58981684(79.72%)	519411(0.7%)	29616864(40.03%)	29364820(39.69%)	29472330(39.84%)	29509354(39.89%)	23976052(32.41%)	35005632(47.32%)	55696296(75.28%)
8_dpi_1	64430044	50647510(78.61%)	50081547(77.73%)	565963(0.88%)	25182242(39.08%)	24899305(38.65%)	25027874(38.85%)	25053673(38.89%)	20095961(31.19%)	29985586(46.54%)	47029620(72.99%)
8_dpi_2	58860648	46547934(79.08%)	46013482(78.17%)	534452(0.91%)	23045895(39.15%)	22967587(39.02%)	22992907(39.06%)	23020575(39.11%)	19048272(32.36%)	26965210(45.81%)	43393530(73.72%)
8_dpi_3	67623462	54077914(79.97%)	53431054(79.01%)	646860(0.96%)	26768402(39.58%)	26662652(39.43%)	26701830(39.49%)	26729224(39.53%)	21720099(32.12%)	31710955(46.89%)	50343984(74.45%)

SUPPLEMENTARY TABLE S5 | Top 100 co-upregulated DEGs.

gene_id	GT FPKM	4 dpi FPKM	6 dpi FPKM	8 dpi FPKM	4 dpi vs GT log2FoldChange	4 dpi vs GT padj	6 dpi vs GT log2FoldChange	6 dpi vs GT padj	8 dpi vs GT log2FoldChange	8 dpi vs GT padj	gene_description	SignalP
PTTG_25256	0.0	1092.9	977.2	39.0	16.2	4.14E-21	16.0	1.21E-27	11.4	1.10E-11	hypothetical protein	SP
PTTG_27333	0.0	8658.5	4382.1	4800.8	15.9	8.05E-27	14.9	4.99E-24	15.1	9.52E-25	hypothetical protein	No SP
PTTG_05620	0.0	691.4	50.9	69.4	15.0	2.50E-24	11.3	4.91E-10	11.7	2.03E-15	hypothetical protein	SP
PTTG_27471	0.0	1063.4	235.9	142.4	14.8	1.55E-23	12.7	4.61E-16	11.9	5.22E-16	hypothetical protein	SP
PTTG_06270	0.0	607.4	777.5	893.7	14.7	1.82E-23	15.0	5.74E-25	15.3	1.15E-25	hypothetical protein	SP
PTTG_12701	0.0	219.1	146.6	139.7	14.6	5.86E-23	14.0	1.57E-21	13.9	2.21E-21	hypothetical protein	SP
PTTG_07988	0.0	205.5	32.3	31.9	14.6	1.57E-22	11.9	8.01E-16	11.9	1.51E-14	hypothetical protein	No SP
PTTG_03405	0.0	314.9	223.4	210.4	14.3	2.77E-22	13.8	5.27E-21	13.8	6.70E-21	hypothetical protein	SP
PTTG_07543	0.0	252.2	18.0	22.2	14.1	1.67E-21	10.3	1.46E-08	10.6	7.93E-13	hypothetical protein	SP
PTTG_00528	0.0	233.5	361.4	37.7	13.7	1.13E-15	14.3	1.51E-22	11.1	1.05E-12	hypothetical protein	No SP
PTTG_07673	0.0	59.0	7.3	9.3	13.7	2.03E-20	10.7	3.32E-10	11.1	9.59E-14	hypothetical protein	SP
PTTG_08629	0.0	233.0	20.4	30.3	13.6	5.13E-20	10.1	2.76E-09	10.7	5.19E-13	hypothetical protein	SP
PTTG_01527	0.0	96.0	106.1	90.4	13.6	5.22E-20	13.7	1.73E-20	13.5	4.02E-20	hypothetical protein	SP
PTTG_28204	0.0	110.0	40.8	25.5	13.5	9.03E-20	12.0	3.14E-16	11.4	1.25E-14	hypothetical protein	No SP
PTTG_27521	0.0	513.3	65.3	57.4	13.4	1.33E-19	10.4	1.21E-09	10.3	4.33E-12	hypothetical protein	SP
PTTG_05844	0.0	152.5	9.7	12.7	13.3	3.04E-19	9.3	2.47E-07	9.7	7.38E-11	hypothetical protein	SP
PTTG_10271	0.3	3112.8	130.5	265.6	13.3	5.19E-164	8.7	4.02E-13	9.7	4.58E-88	hypothetical protein	No SP
novel.1740	0.0	74.1	7.7	5.9	13.2	5.34E-19	9.9	6.71E-11	9.6	1.36E-10	hypothetical protein	No SP
PTTG_05544	0.1	486.7	1263.1	159.6	13.2	2.36E-35	14.6	3.96E-45	11.6	1.66E-28	hypothetical protein	SP
PTTG_27959	0.0	187.5	14.2	16.0	13.1	1.45E-18	9.4	3.67E-07	9.6	1.23E-10	hypothetical protein	SP
PTTG_25812	0.2	1447.9	100.6	169.5	13.1	7.62E-19	9.3	1.33E-07	10.0	9.09E-12	hypothetical protein	SP
novel807	0.0	339.8	38.3	67.5	13.1	1.06E-18	9.9	2.31E-11	10.8	3.14E-13	hypothetical protein	No SP
novel197	0.0	399.6	61.8	65.4	13.1	3.90E-35	10.3	4.67E-22	10.4	1.09E-23	hypothetical protein	SP

novel1181	0.0	194.3	15.8	20.9	13.1	1.48E-18	9.4	6.64E-08	9.8	4.08E-11	hypothetical protein	SP
PTTG_09449	0.0	60.3	39.7	39.2	13.0	4.16E-18	12.4	3.52E-16	12.4	3.74E-17	hypothetical protein	SP
PTTG_26219	0.0	343.7	24.9	34.1	13.0	3.30E-18	9.2	1.38E-07	9.7	7.33E-11	hypothetical protein	SP
PTTG_27354	0.0	338.2	24.5	35.7	13.0	3.32E-18	9.2	5.64E-07	9.8	5.99E-11	hypothetical protein	SP
PTTG_27510	0.0	83.7	37.5	16.9	13.0	8.37E-18	11.8	3.71E-14	10.7	5.41E-13	hypothetical protein	SP
PTTG_10298	0.0	206.4	16.2	22.0	13.0	4.31E-18	9.3	2.11E-07	9.7	6.03E-11	hypothetical protein	SP
novel.406	0.0	68.7	32.8	32.5	12.9	4.33E-18	11.8	1.60E-15	11.8	9.38E-16	hypothetical protein	No SP
PTTG_11744	0.0	37.4	67.1	45.7	12.9	4.46E-18	13.7	6.72E-18	13.2	1.29E-18	hypothetical protein	SP
PTTG_26041	0.0	74.1	12.2	20.9	12.9	4.74E-18	10.3	8.43E-12	11.0	7.87E-14	Collagen	No SP
PTTG_03945	0.0	39.4	19.8	17.9	12.8	1.49E-17	11.8	2.18E-15	11.7	2.53E-15	SET	No SP
PTTG_29948	0.0	163.1	11.8	15.6	12.8	1.35E-17	9.0	5.00E-07	9.4	3.55E-10	hypothetical protein	No SP
PTTG_11707	0.0	93.8	43.4	48.3	12.7	1.65E-17	11.6	5.04E-15	11.8	1.54E-15	hypothetical protein	No SP
PTTG_07125	0.0	50.6	21.9	17.1	12.7	2.20E-17	11.5	1.09E-14	11.1	4.85E-14	hypothetical protein	No SP
PTTG_25232	0.1	368.6	29.3	41.8	12.7	1.03E-17	9.0	5.78E-09	9.5	1.08E-10	hypothetical protein	No SP
PTTG_01095	0.0	83.3	41.0	23.8	12.6	2.24E-17	11.6	4.10E-15	10.8	2.51E-13	orotidine 5'-phosphate decarboxylase	No SP
PTTG_27728	0.0	46.3	26.9	30.9	12.6	2.60E-17	11.8	1.22E-15	12.0	3.11E-16	hypothetical protein	No SP
PTTG_28975	0.0	76.1	45.4	49.5	12.6	2.71E-17	11.8	1.03E-15	12.0	4.56E-16	hypothetical protein	SP
novel.2003	0.0	64.9	25.5	29.8	12.6	3.92E-12	11.2	4.40E-14	11.5	1.06E-13	hypothetical protein	No SP
PTTG_12725	0.0	125.7	12.5	19.1	12.6	3.04E-17	9.2	2.25E-09	9.9	3.64E-11	hypothetical protein	No SP
PTTG_28380	0.0	252.2	87.5	107.1	12.6	8.36E-17	11.0	1.39E-13	11.3	1.59E-14	hypothetical protein	No SP
PTTG_12208	0.0	67.6	21.9	16.4	12.5	6.11E-17	10.9	2.52E-13	10.5	1.43E-12	hypothetical protein	No SP
PTTG_06717	0.0	50.3	8.6	9.6	12.5	4.14E-17	10.0	3.72E-11	10.1	9.68E-12	hypothetical protein	No SP
PTTG_12548	0.0	93.5	13.4	19.2	12.5	4.36E-17	9.7	1.10E-10	10.2	5.08E-12	hypothetical protein	No SP
PTTG_09706	0.0	98.7	10.4	16.6	12.5	4.70E-17	9.2	2.93E-09	9.9	2.77E-11	hypothetical protein	No SP
PTTG_08468	0.2	1029.7	5951.1	1627.5	12.5	3.03E-71	15.0	2.80E-116	13.2	2.33E-90	hypothetical protein	SP
PTTG_03249	0.0	89.1	56.1	191.1	12.5	1.15E-16	11.8	1.15E-15	13.6	1.99E-20	hypothetical protein	SP

PTTG_00174	0.0	57.8	6.2	9.1	12.5	5.53E-17	9.2	7.48E-08	9.8	4.15E-11	hypothetical protein	No SP
PTTG_12066	0.0	186.1	154.5	145.0	12.5	7.65E-17	12.2	1.42E-16	12.1	2.07E-16	hypothetical protein	SP
PTTG_08542	0.0	48.4	5.8	7.7	12.5	5.77E-17	9.4	1.27E-09	9.8	4.88E-11	hypothetical protein	No SP
novel.1707	0.0	48.7	23.2	23.1	12.5	1.00E-16	11.4	1.61E-14	11.4	1.53E-14	hypothetical protein	No SP
PTTG_10519	0.0	44.6	29.8	28.8	12.4	1.28E-16	11.8	1.09E-15	11.8	1.21E-15	hypothetical protein	No SP
PTTG_27401	0.0	115.1	39.9	25.9	12.4	1.29E-16	10.9	4.01E-13	10.2	5.51E-12	hypothetical protein	No SP
PTTG_00522	0.1	584.1	427.9	15.7	12.4	1.04E-42	11.9	2.30E-43	7.2	8.04E-10	hypothetical protein	No SP
PTTG_12184	0.0	56.1	9.5	11.6	12.4	1.22E-16	9.8	7.90E-11	10.1	1.02E-11	hypothetical protein	No SP
novel.562	0.0	68.8	8.1	7.3	12.3	1.77E-16	9.2	1.64E-09	9.1	1.23E-09	hypothetical protein	No SP
PTTG_08305	0.0	36.4	15.3	13.6	12.3	1.31E-16	11.1	8.33E-14	10.9	1.40E-13	hypothetical protein	No SP
PTTG_11740	0.1	295.5	230.3	170.4	12.3	8.20E-32	12.0	1.19E-30	11.5	1.05E-28	hypothetical protein	No SP
PTTG_07972	0.0	73.8	33.3	30.7	12.3	2.00E-16	11.2	4.73E-14	11.1	7.21E-14	hypothetical protein	No SP
PTTG_01596	0.0	43.7	3.9	4.9	12.3	8.24E-16	8.8	1.46E-08	9.2	8.07E-10	MFS_1	No SP
PTTG_01252	0.2	834.5	2312.8	2624.0	12.3	4.72E-11	13.8	6.51E-136	14.0	8.38E-140	protein NMT1	No SP
PTTG_26988	0.0	63.9	3.1	4.2	12.3	2.16E-16	7.9	7.95E-07	8.4	3.16E-08	hypothetical protein	No SP
PTTG_11745	0.0	24.9	21.4	17.8	12.3	2.31E-16	12.0	6.40E-16	11.8	1.58E-15	hypothetical protein	No SP
PTTG_06182	0.0	27.3	178.4	134.5	12.2	3.45E-16	14.9	4.29E-24	14.5	4.11E-23	hypothetical protein	No SP
PTTG_02710	0.0	93.9	215.1	157.8	12.2	1.17E-16	13.4	4.35E-20	13.0	6.75E-19	Cys_Met_Meta_PP	No SP
PTTG_06906	0.0	25.2	2.2	2.7	12.2	6.66E-16	8.7	3.57E-08	9.0	1.96E-09	hypothetical protein	No SP
PTTG_08879	0.0	35.2	22.5	25.4	12.2	6.46E-16	11.5	6.60E-15	11.7	2.14E-15	AA_permease	No SP
novel.1399	0.0	48.7	2.0	3.5	12.2	4.06E-16	7.6	1.47E-06	8.4	2.94E-08	hypothetical protein	No SP
PTTG_28256	0.0	78.1	16.7	17.3	12.2	6.62E-16	9.9	4.48E-11	10.0	1.80E-11	hypothetical protein	No SP
PTTG_01085	0.0	27.5	6.2	6.8	12.1	5.28E-16	10.0	2.83E-11	10.1	1.23E-11	hypothetical protein	No SP
PTTG_27353	0.0	178.9	15.2	19.7	12.1	1.44E-15	8.5	5.27E-08	8.9	2.49E-09	hypothetical protein	No SP
PTTG_05441	0.0	78.0	173.9	148.9	12.1	4.53E-16	13.2	2.84E-19	13.0	5.78E-19	Sugar_tr	No SP
PTTG_01087	0.0	25.1	2.8	3.7	12.0	1.08E-15	8.8	3.60E-07	9.3	5.02E-10	hypothetical protein	No SP

PTTG_12594	0.0	26.8	14.1	13.1	12.0	1.57E-15	11.1	7.18E-14	11.0	1.16E-13	hypothetical protein	No SP
novel.1687-	0.0	38.4	20.3	20.3	12.0	1.16E-15	11.1	7.46E-14	11.1	6.71E-14	hypothetical protein	No SP
PTTG_03152	0.0	186.0	32.3	35.8	12.0	2.21E-30	9.5	5.18E-19	9.6	2.76E-20	hypothetical protein	No SP
PTTG_11755	0.0	56.3	22.6	13.0	12.0	1.16E-15	10.7	7.07E-13	9.9	3.25E-11	Ferric_reduct	No SP
PTTG_12002	0.0	28.4	12.3	11.5	11.9	2.36E-15	10.7	7.01E-13	10.6	7.05E-13	hypothetical protein	No SP
PTTG_12004	0.0	38.1	15.4	13.3	11.9	1.79E-15	10.6	9.47E-13	10.4	2.29E-12	hypothetical protein	No SP
PTTG_12769	0.0	46.2	4.7	5.8	11.9	2.27E-15	8.6	6.03E-08	8.9	2.42E-09	hypothetical protein	No SP
PTTG_27172	0.0	68.0	32.5	23.4	11.9	3.28E-15	10.8	4.15E-13	10.3	3.15E-12	hypothetical protein	No SP
PTTG_29929	0.0	41.2	5.8	8.3	11.9	2.31E-15	9.0	3.28E-09	9.6	1.36E-10	hypothetical protein	No SP
PTTG_04622	0.0	25.9	13.6	19.1	11.8	3.56E-15	10.9	1.69E-13	11.4	1.06E-14	hypothetical protein	No SP
PTTG_12723	0.0	36.8	12.0	10.0	11.8	6.93E-15	10.2	8.15E-12	9.9	2.00E-11	hypothetical protein	No SP
PTTG_12348	0.0	31.2	18.4	21.8	11.8	3.31E-15	11.0	1.11E-13	11.3	1.89E-14	hypothetical protein	No SP
PTTG_26831	0.0	223.5	207.5	201.4	11.8	5.84E-15	11.7	2.36E-15	11.7	2.56E-15	hypothetical protein	No SP
PTTG_12568	0.0	142.1	11.5	13.2	11.8	5.74E-15	8.1	1.29E-07	8.3	2.96E-08	hypothetical protein	No SP
novel.1235	0.0	49.9	19.9	26.5	11.7	6.29E-15	10.4	2.75E-12	10.8	2.54E-13	hypothetical protein	No SP
PTTG_11940	0.0	35.2	139.7	36.7	11.7	4.23E-15	13.7	6.68E-21	11.8	9.57E-16	hypothetical protein	No SP
PTTG_03185	0.0	29.2	22.0	17.0	11.7	1.97E-14	11.3	2.82E-14	10.9	1.62E-13	hypothetical protein	No SP
PTTG_12474	0.0	52.8	7.3	9.7	11.7	9.15E-15	8.8	8.34E-09	9.2	7.43E-10	hypothetical protein	No SP
novel.256	0.0	33.6	6.9	7.1	11.7	1.38E-14	9.4	1.28E-09	9.4	2.44E-10	hypothetical protein	No SP
PTTG_26601	0.0	34.4	56.6	59.1	11.7	2.42E-14	12.4	4.93E-17	12.4	2.97E-17	hypothetical protein	SP
PTTG_28724	0.0	20.1	2.1	3.3	11.7	7.94E-15	8.4	1.20E-07	9.0	1.57E-09	hypothetical protein	No SP
PTTG_12231	0.0	56.2	29.4	33.1	11.7	2.30E-14	10.7	5.14E-13	10.9	1.78E-13	hypothetical protein	No SP
novel.1754	0.0	41.6	21.9	25.0	11.6	1.03E-14	10.7	4.88E-13	10.9	1.69E-13	hypothetical protein	No SP
novel.563	0.0	36.5	18.3	13.4	11.6	1.66E-14	10.6	8.63E-13	10.2	7.60E-12	hypothetical protein	No SP

SUPPLEMENTARY TABLE S6 | Top 100 co-downregulated DEGs.

gene_id	GT FPKM	4 dpi FPKM	6 dpi FPKM	8 dpi FPKM	4 dpi vs GT log2FoldChange	4 dpi vs GT padj	6 dpi vs GT log2FoldChange	6 dpi vs GT padj	8 dpi vs GT log2FoldChange	8 dpi vs GT padj	gene_description
novel.770	141.3	0.1	12.2	13.9	-11.0	8.96E-15	-3.5	6.96E-98	-3.3	4.03E-184	hypothetical protein
PTTG_12720	30.9	0.0	0.0	1.2	-10.9	3.38E-13	-9.9	1.60E-59	-4.6	7.72E-174	hypothetical protein
novel.1685	26.6	0.0	0.4	2.5	-10.5	2.65E-12	-6.1	1.06E-153	-3.4	7.80E-136	hypothetical protein
PTTG_28793	105.2	0.1	1.0	12.1	-10.2	5.04E-13	-6.7	1.02E-277	-3.1	5.32E-98	hypothetical protein
PTTG_29556	39.3	0.0	2.2	3.6	-9.8	9.60E-11	-4.1	1.56E-69	-3.4	1.15E-92	hypothetical protein
PTTG_25387	9.8	0.0	0.2	0.9	-9.4	6.63E-10	-5.6	8.49E-88	-3.4	1.80E-57	hypothetical protein
PTTG_27330	245.4	0.4	0.4	0.6	-9.2	2.05E-59	-9.3	0	-8.7	0	hypothetical protein
novel.640	41.1	0.0	0.2	2.0	-9.0	4.97E-09	-7.3	9.52E-46	-4.3	2.14E-10	hypothetical protein
PTTG_27458	431.5	0.8	0.3	4.7	-9.0	5.23E-13	-10.3	2.59E-131	-6.5	0	hypothetical protein
PTTG_28830	13.4	0.0	0.8	0.8	-8.9	8.44E-09	-4.0	1.70E-55	-4.0	1.33E-67	hypothetical protein
PTTG_04752	129.9	0.3	0.1	1.1	-8.8	1.12E-54	-10.0	2.07E-236	-6.9	0	hypothetical protein
PTTG_12496	497.2	1.1	0.7	9.0	-8.8	1.27E-187	-9.5	2.15E-77	-5.8	0	Abhydrolase_2
PTTG_12292	37.2	0.1	4.4	6.3	-8.7	2.35E-16	-3.1	1.34E-60	-2.5	1.92E-117	hypothetical protein
PTTG_02548	2465.5	6.1	1.2	4.6	-8.7	0	-10.9	2.44E-31	-9.0	0	hypothetical protein
PTTG_12435	5314.1	13.8	6.8	106.2	-8.6	0	-9.6	1.34E-165	-5.6	0	hypothetical protein
PTTG_26026	23.2	0.0	0.1	2.2	-8.5	4.21E-08	-8.1	9.02E-26	-3.4	9.94E-38	hypothetical protein
PTTG_31124	5991.3	16.7	81.1	467.1	-8.5	1.21E-295	-6.2	1.76E-281	-3.6	0	hypothetical protein
PTTG_06135	672.1	1.9	3.6	17.4	-8.5	2.33E-303	-7.5	0	-5.2	0	hypothetical protein
PTTG_29261	806.1	2.3	1.3	24.9	-8.5	6.54E-57	-9.2	8.63E-50	-5.0	0	hypothetical protein
PTTG_08941	7103.1	21.8	4.1	123.4	-8.3	1.92E-59	-10.7	7.27E-41	-5.8	0	hypothetical protein
PTTG_12086	563.0	1.7	0.8	26.5	-8.3	4.07E-191	-9.4	4.26E-60	-4.4	7.38E-277	hypothetical protein
PTTG_11639	4751.5	15.2	5.3	148.7	-8.3	0	-9.8	3.33E-105	-5.0	0	hypothetical protein
PTTG_03367	1140.8	3.7	1.7	5.7	-8.3	0	-9.4	1.42E-69	-7.6	0	hypothetical protein

PTTG_05984	809.2	2.6	2.1	12.9	-8.3	0	-8.6	0	-5.9	0	hypothetical protein
PTTG_09130	1292.9	4.2	1.1	26.9	-8.3	0	-10.2	1.68E-78	-5.6	0	hypothetical protein
novel.276	14.0	0.0	0.0	0.0	-8.3	1.45E-07	-10.7	2.92E-18	-9.7	1.44E-15	hypothetical protein
PTTG_02563	241.3	0.7	1.7	3.8	-8.2	2.75E-181	-7.2	0	-6.0	0	Na_H_Exchanger
PTTG_01246	1968.1	6.6	1.5	57.3	-8.2	0	-10.4	1.67E-55	-5.1	0	hypothetical protein
PTTG_25386	13548.2	44.7	9.8	206.6	-8.2	0	-10.4	4.34E-35	-6.0	0	hypothetical protein
PTTG_12434	221.1	0.7	1.0	6.6	-8.2	7.22E-101	-7.8	0	-5.0	0	Asp
PTTG_02667	2510.0	8.4	2.6	74.5	-8.2	0	-9.9	4.18E-68	-5.0	0	hypothetical protein
PTTG_02477	1030.0	3.5	4.8	34.4	-8.2	0	-7.7	0	-4.9	0	hypothetical protein
novel.1959	8.3	0.0	0.1	0.5	-8.2	2.11E-07	-6.2	7.27E-36	-3.9	8.05E-43	hypothetical protein
PTTG_11882	364.8	1.2	0.9	11.1	-8.2	1.28E-166	-8.6	2.14E-64	-5.0	0	hypothetical protein
PTTG_29552	14.5	0.0	0.2	1.3	-8.1	2.69E-07	-6.3	1.71E-32	-3.5	4.44E-36	hypothetical protein
PTTG_29176	9.4	0.0	0.1	0.2	-8.1	2.65E-07	-7.4	1.01E-25	-5.3	2.17E-42	hypothetical protein
PTTG_10214	295.1	1.0	1.6	14.0	-8.1	1.64E-105	-7.5	0	-4.4	0	hypothetical protein
PTTG_25925	55.9	0.0	1.3	2.4	-8.1	2.89E-07	-5.5	1.03E-38	-4.5	9.00E-36	hypothetical protein
PTTG_04172	2036.8	7.5	2.9	59.0	-8.1	0	-9.5	3.28E-154	-5.1	0	hypothetical protein
PTTG_04309	331.0	1.2	19.8	53.8	-8.0	8.35E-136	-4.0	1.98E-171	-2.6	3.77E-168	hypothetical protein
PTTG_00068	1041.4	3.8	1.3	45.2	-8.0	0	-9.6	5.89E-167	-4.5	6.88E-245	Polysacc_deac_1
PTTG_28316	101.3	0.4	0.9	2.6	-8.0	1.35E-32	-6.7	3.02E-278	-5.3	0	hypothetical protein
PTTG_11955	57.8	0.2	0.9	4.5	-8.0	1.40E-56	-6.1	0	-3.7	2.89E-201	hypothetical protein
PTTG_06229	928.7	3.6	0.6	18.5	-8.0	0	-10.5	7.18E-68	-5.6	0	hypothetical protein
PTTG_04559	61.2	0.2	0.1	0.7	-8.0	4.40E-20	-8.8	3.34E-103	-6.3	1.23E-228	Abhydrolase_1
PTTG_28485	226.4	0.9	1.3	13.4	-8.0	2.13E-79	-7.5	0	-4.0	4.86E-277	hypothetical protein
PTTG_03800	12980.5	50.0	10.4	178.8	-8.0	0	-10.3	4.14E-39	-6.1	0	hypothetical protein
PTTG_07374	673.4	2.6	2.1	25.4	-8.0	1.17E-287	-8.3	0	-4.7	0	hypothetical protein
PTTG_27099	11561.2	44.6	192.9	1015.6	-8.0	0	-5.9	3.07E-241	-3.5	0	hypothetical protein

PTTG_28961	697.8	2.8	0.7	2.6	-8.0	3.01E-205	-10.0	0	-8.0	0	hypothetical protein
PTTG_00474	1246.4	4.9	2.0	38.6	-8.0	0	-9.2	3.44E-107	-5.0	0	hypothetical protein
PTTG_00275	19791.1	77.3	53.7	1329.9	-8.0	0	-8.5	2.98E-96	-3.9	1.21E-212	hypothetical protein
PTTG_00665	273.4	1.1	1.6	11.1	-7.9	1.99E-121	-7.4	0	-4.6	0	hypothetical protein
PTTG_25763	35.0	0.1	0.1	0.1	-7.9	5.07E-08	-8.4	2.73E-41	-8.0	2.00E-49	hypothetical protein
PTTG_12583	476.0	2.0	0.5	7.6	-7.9	1.28E-202	-10.0	0	-5.9	0	hypothetical protein
PTTG_04011	1208.8	5.1	3.2	43.6	-7.9	0	-8.5	0	-4.8	0	hypothetical protein
PTTG_06084	5953.0	25.4	7.4	266.5	-7.9	0	-9.6	6.34E-53	-4.4	0	hypothetical protein
PTTG_12575	668.1	3.0	10.6	49.9	-7.9	8.47E-230	-6.0	1.18E-42	-3.7	5.55E-250	hypothetical protein
PTTG_01670	424.3	1.9	1.2	24.0	-7.8	1.90E-230	-8.5	4.34E-97	-4.1	1.47E-239	hypothetical protein
PTTG_26898	94.9	0.4	0.1	2.3	-7.8	3.53E-65	-9.8	4.88E-29	-5.3	7.98E-74	zf-C2H2
PTTG_06712	2131.8	9.2	12.0	158.5	-7.8	0	-7.5	0	-3.7	1.83E-190	Peptidase_S8
PTTG_07626	3074.5	13.2	15.3	180.6	-7.8	0	-7.6	0	-4.1	2.04E-261	hypothetical protein
PTTG_02225	4386.5	19.3	6.7	151.5	-7.8	0	-9.3	1.39E-63	-4.8	2.90E-306	hypothetical protein
PTTG_02236	1189.5	5.1	1.6	54.8	-7.8	0	-9.5	7.93E-62	-4.4	0	hypothetical protein
novel.250	9.4	0.1	0.4	0.9	-7.8	3.65E-13	-4.5	2.78E-89	-3.3	2.76E-101	PIF1-like helicase
PTTG_27669	10118.8	44.8	120.4	1257.4	-7.8	0	-6.4	0	-3.0	1.17E-232	hypothetical protein
PTTG_07558	535.9	2.4	0.4	2.9	-7.8	1.44E-132	-10.4	1.26E-43	-7.5	0	hypothetical protein
PTTG_09270	10958.9	49.0	139.8	1290.0	-7.8	0	-6.3	0	-3.1	5.93E-236	hypothetical protein
PTTG_06192	372.2	1.7	1.3	10.6	-7.8	1.12E-41	-8.1	1.67E-93	-5.1	0	hypothetical protein
PTTG_09713	45.8	0.3	1.4	3.3	-7.8	5.07E-13	-5.1	1.08E-134	-3.7	3.83E-105	hypothetical protein
PTTG_09156	10355.0	46.8	28.3	811.3	-7.8	0	-8.5	1.21E-68	-3.6	3.76E-205	hypothetical protein
PTTG_27630	116.0	0.5	1.1	1.2	-7.8	2.07E-69	-6.7	0	-6.5	0	L-ascorbate oxidase
PTTG_00152	6051.9	27.6	50.2	369.9	-7.8	0	-6.9	0	-4.0	0	hypothetical protein
PTTG_04502	165.9	0.8	2.2	12.1	-7.8	1.69E-144	-6.2	0	-3.7	3.60E-294	hypothetical protein
PTTG_12685	103.3	0.5	0.2	9.9	-7.8	6.67E-24	-9.3	1.47E-43	-3.4	2.78E-120	zf-C2H2

PTTG_01601	92.4	0.4	6.0	17.9	-7.7	4.42E-30	-3.9	4.80E-218	-2.3	4.60E-72	hypothetical protein
PTTG_01334	11434.6	52.7	175.7	1266.3	-7.7	0	-6.0	0	-3.1	6.20E-228	hypothetical protein
PTTG_07455	85.6	0.4	0.3	1.4	-7.7	2.28E-24	-8.4	2.59E-129	-5.9	9.93E-61	hypothetical protein
PTTG_12290	30.8	0.2	0.5	2.8	-7.7	1.50E-07	-5.8	3.33E-60	-3.4	1.81E-45	hypothetical protein
PTTG_08962	4009.0	18.9	7.2	258.3	-7.7	0	-9.1	4.27E-43	-3.9	4.09E-208	hypothetical protein
PTTG_04054	598.1	3.0	0.6	1.6	-7.7	8.37E-191	-10.1	1.44E-59	-8.5	0	hypothetical protein
novel.192	4.7	0.0	0.0	0.1	-7.7	1.63E-06	-9.2	1.40E-13	-5.1	4.77E-07	Reverse transcriptase
PTTG_01007	201.6	1.0	0.2	7.0	-7.7	1.15E-171	-9.8	3.48E-17	-4.8	0	hypothetical protein
PTTG_08292	76.8	0.4	0.2	2.5	-7.7	8.41E-30	-8.4	5.34E-158	-4.9	1.12E-302	hypothetical protein
PTTG_28414	709.5	3.4	1.2	29.8	-7.7	2.92E-304	-9.2	0	-4.5	3.33E-254	hypothetical protein
PTTG_04998	327.9	1.5	3.0	18.5	-7.7	8.51E-170	-6.7	0	-4.1	1.64E-254	hypothetical protein
PTTG_26873	585.8	3.0	1.1	27.9	-7.7	1.07E-168	-9.0	1.24E-98	-4.4	2.91E-65	hypothetical protein
PTTG_04707	2443.6	11.9	3.4	88.1	-7.7	0	-9.5	3.67E-40	-4.8	1.38E-283	hypothetical protein
PTTG_07714	24134.7	116.6	72.1	1458.5	-7.7	0	-8.4	4.74E-115	-4.0	1.87E-203	hypothetical protein
PTTG_04712	1042.1	5.3	1.0	0.6	-7.7	4.70E-245	-10.1	2.10E-149	-10.8	0	hypothetical protein
PTTG_08472	124.8	0.6	0.8	1.3	-7.7	5.30E-102	-7.3	0	-6.6	0	hypothetical protein
PTTG_29284	375.2	1.8	1.1	15.5	-7.7	3.28E-82	-8.4	0	-4.6	0	hypothetical protein
PTTG_04820	120.6	0.6	3.3	3.9	-7.7	5.66E-46	-5.2	6.17E-274	-4.9	0	hypothetical protein
PTTG_29042	120.5	0.6	2.1	9.5	-7.7	1.22E-56	-5.8	0	-3.6	4.57E-216	hypothetical protein
PTTG_31003	39.0	0.0	1.5	8.3	-7.6	2.97E-06	-4.6	0.001643943	-2.2	5.31E-14	hypothetical protein
PTTG_11693	8847.7	44.6	46.0	164.5	-7.6	0	-7.6	0	-5.7	0	hypothetical protein
PTTG_09298	460.5	2.5	0.4	15.9	-7.6	1.73E-156	-10.2	1.96E-51	-4.8	0	hypothetical protein
PTTG_00399	2392.4	12.1	100.8	368.9	-7.6	0	-4.5	0	-2.7	1.99E-254	hypothetical protein
PTTG_08532	6.4	0.0	0.3	1.2	-7.6	2.19E-07	-4.4	2.46E-67	-2.3	1.49E-30	hypothetical protein

SUPPLEMENTARY TABLE S7 | Predicted effectors encoded by the co-upregulated DEGs.

PTTG accession	EffectorP probability	Conserved domain (pfam)	GT FPKM	4 dpi FPKM	6 dpi FPKM	8 dpi FPKM
PTTG_27310	1.00	N.A.	0.2	184.4	26.2	33.0
PTTG_26436	1.00	Protein kinase domain (PF00069)	25.8	86.9	95.5	81.9
PTTG_07398	1.00	Thaumatin (PF00314)	3.6	62.6	763.6	386.1
PTTG_00671	1.00	N.A.	0.6	12.3	3.5	3.9
novel.559	1.00	N.A.	0.0	4.5	1.3	1.2
novel.492	1.00	N.A.	0.0	6.9	3.1	3.9
PTTG_07345	0.99	N.A.	3.4	116.9	11.6	15.2
novel.1420	0.99	N.A.	0.0	4.6	0.5	0.9
novel.1021	0.98	N.A.	0.0	4.0	0.8	0.7
PTTG_25283	0.98	N.A.	0.0	14.1	3.9	3.6
novel.211	0.97	N.A.	0.3	0.7	1.2	1.1
PTTG_29714	0.97	Procyclic acidic repetitive protein (PF05887)	0.1	12.2	1.2	1.9
PTTG_04629	0.97	N.A.	0.6	57.0	3.8	6.3
novel.1545	0.97	N.A.	0.0	5.4	1.4	1.2
PTTG_00522	0.97	N.A.	0.1	584.1	428.0	15.7
novel.467	0.97	N.A.	0.0	4.7	1.3	2.5
PTTG_25256	0.96	N.A.	0.0	1092.9	977.2	39.0
PTTG_09007	0.96	N.A.	0.4	115.1	10.2	13.1
PTTG_05834	0.96	N.A.	2.2	274.9	29.3	34.8
PTTG_30152	0.95	N.A.	0.1	66.6	6.0	8.5
PTTG_05844	0.95	N.A.	0.0	152.5	9.7	12.7
novel.563	0.95	N.A.	0.0	36.6	18.3	13.4
novel.1680	0.95	N.A.	0.3	4.0	4.1	5.1
PTTG_00528	0.94	N.A.	0.0	233.5	361.4	37.7
novel.1540	0.94	N.A.	0.0	4.8	0.7	1.4
PTTG_28461	0.94	N.A.	3.0	28.4	30.2	26.3
PTTG_28742	0.94	Protein of unknown function DUF3430 (PF11912)	1.4	4.3	8.7	8.7
PTTG_01781	0.94	N.A.	0.5	68.2	8.6	10.8
PTTG_11983	0.93	Ligase Pab1020 C-terminal region (PF18330)	0.0	15.0	8.4	4.8
PTTG_00114	0.93	N.A.	2.6	168.8	118.7	67.7
novel.697	0.93	N.A.	0.0	1.9	3.0	3.6
PTTG_03570	0.92	N.A.	0.7	569.6	61.3	80.4
PTTG_03780	0.92	N.A.	0.2	169.4	9.5	15.7
PTTG_11722	0.92	Apoptosis regulator M11L like (PF11099)	0.0	29.0	3.7	5.2
PTTG_12651	0.92	N.A.	0.0	11.5	14.6	19.4
PTTG_02291	0.91	N.A.	0.1	16.8	1.3	2.3
PTTG_11720	0.89	N.A.	0.2	22.7	3.5	4.7
novel.1404	0.89	N.A.	0.0	1.6	1.5	0.9
PTTG_05290	0.89	N.A.	0.2	298.4	24.5	32.0
novel.637	0.89	N.A.	0.0	1.6	0.5	1.9
novel.1443	0.89	N.A.	0.0	2.8	2.3	3.1

novel.1252	0.89	N.A.	0.0	2.5	0.4	0.7
PTTG_05614	0.88	N.A.	1.3	756.9	61.4	83.9
PTTG_27631	0.88	N.A.	0.5	21.2	2.4	3.7
PTTG_28226	0.88	Low iron-inducible periplasmic protein (PF07692)	2.1	446.7	33.7	54.1
PTTG_09419	0.88	Myticin pre-proprotein from the mussel (PF10690)	0.1	8.8	5.0	3.3
PTTG_11938	0.87	N.A.	0.0	26.3	2.7	3.2
novel.473	0.87	N.A.	0.0	3.1	0.4	1.5
novel.495	0.86	N.A.	0.0	2.6	2.3	2.2
PTTG_27172	0.86	N.A.	0.0	68.0	32.6	23.4
PTTG_00307	0.86	N.A.	0.3	30.5	77.9	46.9
novel.1931	0.86	N.A.	0.0	0.6	0.9	1.0
PTTG_03497	0.85	N.A.	0.3	79.0	83.4	58.7
PTTG_29644	0.84	N.A.	15.7	791.9	69.8	87.2
novel.471	0.84	N.A.	0.0	1.9	0.4	0.5
PTTG_12427	0.82	N.A.	0.0	11.0	6.4	7.0
novel.343	0.82	N.A.	0.0	1.7	1.7	1.9
novel.472	0.81	N.A.	0.0	5.0	0.9	1.7
novel.1122	0.81	N.A.	0.0	18.3	4.3	4.1
PTTG_02768	0.81	FAT domain (PF02259)	0.1	51.2	55.0	49.4
PTTG_12542	0.78	Protein of unknown function DUF829 (PF05705)	6.5	60.6	17.1	15.8
novel.1543	0.78	N.A.	0.0	4.3	3.9	4.2
novel.1633	0.77	N.A.	0.0	2.0	0.9	1.3
PTTG_11740	0.74	Protein of unknown function DUF333 (PF03891)	0.1	295.5	230.3	170.4
PTTG_03539	0.71	Common central domain of tyrosinase (PF00264)	0.3	10.6	1.2	2.5
PTTG_28700	0.70	N.A.	31.8	516.3	73.8	83.8
novel.92	0.69	N.A.	0.0	4.1	4.6	5.3
PTTG_05620	0.68	N.A.	0.0	691.5	50.9	69.4
PTTG_12319	0.65	N.A.	0.0	17.7	10.7	13.6
novel.626	0.59	N.A.	0.0	9.9	5.1	5.5
novel.377	0.59	N.A.	0.1	1.1	1.0	2.8
novel.846	0.59	N.A.	0.0	1.3	1.9	1.9
PTTG_12711	0.59	N.A.	0.3	40.6	3.2	3.1
PTTG_25324	0.57	N.A.	2.1	11.4	7.9	8.1
PTTG_04890	0.57	N.A.	0.0	5.7	0.7	1.1
PTTG_03168	0.56	N.A.	0.6	344.3	52.9	46.5
PTTG_12701	0.54	N.A.	0.0	219.2	146.6	139.7
PTTG_05870	0.53	Protein of unknown function DUF3455 (PF11937)	0.0	6.8	1.4	1.6
novel.1256	0.50	N.A.	0.0	2.7	1.0	0.6

SUPPLEMENTARY TABLE S8 | *Pt* effector candidates containing identical sequence of RXLR or a complete RXLR-dEER-like motif.

Type	Gene accession	RXLR-dEER feature	Conserved domain (pfam)	EffectorP prediction	Effector probability	GT FPKM	4 dpi FPKM	6 dpi FPKM	8 dpi FPKM
Secreted proteins containing identical sequence of RXLR	PTTG_26187	RALR	Clostridial binary toxin B/anthrax toxin PA domain 2 (PF17475)	Effector	0.839	3.06	38.48	4.67	6.27
	PTTG_25160	RALR	Clostridial binary toxin B/anthrax toxin PA domain 2 (PF17475)	Effector	0.837	11.40	16.55	3.36	5.56
	PTTG_27311	RPLR	N. A.	Effector	0.834	19.84	18.50	174.19	75.57
	PTTG_27487	RQLR	N. A.	Effector	0.668	2.21	0.97	10.18	12.42
	PTTG_03308	RYLR	6-phosphofructo-2-kinase (PF01591)	Non-effector	0.983	28.75	35.21	79.15	81.11
	PTTG_05912	RALR	tRNA synthetase class II core domain (PF00587)	Non-effector	0.971	45.94	28.29	23.49	21.04
	novel.833	RYLR-IEDK	Ring finger domain (PF13639)	Non-effector	0.969	0.00	3.67	1.57	1.77
	novel.861	RSLR	N.A.	Non-effector	0.955	0.00	1.75	1.15	0.76
	PTTG_05946	RSLR	Eukaryotic aspartyl protease (PF00026)	Non-effector	0.936	0.09	7.75	26.82	25.64
	novel.1536	RDLR	N.A.	Non-effector	0.92	0.00	2.86	0.82	1.01
	novel.1332	RVLR	Trehalose-phosphatase (PF02358)	Non-effector	0.905	0.00	3.13	1.67	2.09
	PTTG_07204	RSLR	N.A.	Non-effector	0.889	1.12	118.99	19.70	21.21
	PTTG_06625	RGLR	Attacin N-terminal region (PF03768)	Non-effector	0.887	0.05	9.01	0.85	1.27
	PTTG_05306	RALR	Pectinesterase (PF01095)	Non-effector	0.865	0.85	0.17	1.49	5.14
	PTTG_00017	RALR	Pectinesterase (PF01095)	Non-effector	0.847	0.24	0.37	5.91	15.70
	PTTG_00174	RRLR	N.A.	Non-effector	0.825	0.00	57.78	6.17	9.12
	PTTG_06330	RPLR	N.A.	Non-effector	0.796	0.35	1.50	0.50	0.65
	PTTG_06020	RPLR	Glycosyl hydrolase family 7 (PF00840)	Non-effector	0.788	10.52	63.37	8.63	9.52
	PTTG_06205	RPLR	Abi-like protein (PF07751)	Non-effector	0.741	0.21	11.00	5.62	4.52
	novel.1116	RALR	N.A.	Non-effector	0.731	0.00	0.30	7.20	6.77
Secreted proteins containing a	PTTG_30134	RKIT-DEDK	N.A.	Effector	0.934	0.00	15.64	1.83	2.61
	PTTG_29433	KVLA-YDEK	N.A.	Effector	0.738	0.00	11.93	1.57	2.14

complete RXLR-dEER-like motif	PTTG_12320	RPLL-PEEK	N.A.	Effector	0.64	0.77	8.29	4.82	5.00
	PTTG_12434	KDFQ-TDEK	Eukaryotic aspartyl protease (PF00026)	Non-effector	0.971	221.06	0.72	1.00	6.58
	PTTG_09121	RSIT-PDER	Glycosyl Hydrolase Family 88 (PF07470)	Non-effector	0.911	0.00	8.67	3.05	3.01
	PTTG_30127	RAFF-PDDK	N.A.	Non-effector	0.907	0.00	0.00	0.04	0.02
	novel.6	RYMF-LDEK	N.A.	Non-effector	0.829	0.00	3.44	1.31	1.50
	PTTG_12224	HDLA-IEER	Ring finger domain (PF13639)	Non-effector	0.759	16.07	1.99	4.47	5.30
	PTTG_26127	HLLL-EDER	Peptidase inhibitor I9 (PF05922, insignificant)	Non-effector	0.703	850.83	324.61	330.58	467.76

SUPPLEMENTARY TABLE S9 | Genome-wide prediction of CRN-like effector candidates from the wheat leaf rust fungus.

Accession	CRN feature	EffectorP	Probability	Conserved domain (pfam)	GT FPKM	4 dpi FPKM	6 dpi FPKM	8 dpi FPKM
OAV94948.1	LLLAK	Effector	0.762	SelR domain (PF01641)	N.A.	N.A.	N.A.	N.A.
PTTG_06512	LRFKN	Effector	0.744	Thaumatin (PF00314)	292.6	14.0	41.1	147.1
novel.300	LFFAR	Effector	0.728	N.A.	0.0	0.3	1.7	1.2
PTTG_28213	LFLAN	Effector	0.718	N.A.	3.6	0.5	1.8	2.3
PTTG_28846	LLKAK	Effector	0.674	Cleavage stimulation factor subunit 1, dimerisation domain (PF16699)	0.0	40.4	5.8	5.1
PTTG_02142	LLLKN	Effector	0.598	Polysaccharide deacetylase (PF01522)	0.1	3.9	20.4	167.0
novel.910	LLFRK	Non-effector	0.971	N.A.	0.0	5.4	3.2	2.9
PTTG_02264	LYLKR	Non-effector	0.969	N.A.	2298.9	107.8	51.3	236.5
PTTG_12453	LRKVK	Non-effector	0.969	DDHD domain (PF02862)	0.0	21.1	6.8	5.7
novel.837	LFLKK	Non-effector	0.956	N.A.	0.0	1.1	0.8	1.3
PTTG_07031	LLLAR	Non-effector	0.908	N.A.	0.1	22.3	2.3	3.8
PTTG_03567	LRFAN	Non-effector	0.906	Carboxylesterase family (PF00135)	11.2	1.9	43.3	50.8
PTTG_12337	LLKTR	Non-effector	0.844	N.A.	266.5	85.1	71.1	71.2
PTTG_29375	LFLKN	Non-effector	0.843	N.A.	0.0	0.0	0.0	0.0
PTTG_03055	LFLKN	Non-effector	0.751	N.A.	0.4	1.0	0.6	0.6
PTTG_08596	LLLAN	Non-effector	0.739	Peptidase C13 family (PF01650)	53.4	33.7	32.9	32.2
PTTG_25398	LLFRR	Non-effector	0.571	N.A.	0.0	0.0	0.0	0.0
PTTG_00399	LLLRR	Non-effector	0.509	Lytic transglycolase DPBB_1 (PF03330)	2392.4	12.1	100.8	368.9
PTTG_25292	LRLRN	Unlikely effector	0.502	N.A.	0.0	0.0	0.5	7.3

SUPPLEMENTARY TABLE S10 | Genome-wide prediction of Y/F/WxC effector candidates from the wheat leaf rust fungus.

Gene accession	Y/F/WxC feature	EffectorP prediction	Effector probability	Conserved domain (pfam)	GT FPKM	4 dpi FPKM	6 dpi FPKM	8 dpi FPKM
PTTG_25531	FVC	Effector	0.985	N.A.	4.81	1.05	3.72	4.22
PTTG_25337	YTC	Effector	0.982	N.A.	1.11	0.93	1.14	2.05
PTTG_26924*	FKC	Effector	0.979	N.A.	5.10	0.00	1.83	2.83
PTTG_26924	FkC	Effector	0.979	N.A.	5.10	0.00	1.83	2.83
PTTG_28926	FHC	Effector	0.978	N.A.	1.22	0.67	1.59	1.08
PTTG_27706	FNC	Effector	0.978	N.A.	0.00	0.00	0.00	0.00
PTTG_26781	YRC	Effector	0.978	Kyakuja-Dileera-Zisupton transposase (PF18758)<br>CxC1 like cysteine cluster associated with KDZ transposases (PF18802)	0.00	0.00	0.09	0.10
PTTG_12170*	FGC	Effector	0.973	Saposin A-type domain (PF02199)	0.00	1.14	2.27	1.71
PTTG_27731	FTC	Effector	0.964	N.A.	0.00	18.45	1.59	2.13
PTTG_26049	FKC	Effector	0.963	N.A.	0.00	0.00	0.00	0.00
PTTG_11646	FGC	Effector	0.961	N.A.	4.08	148.33	10.35	15.34
PTTG_07455	FSC	Effector	0.961	N.A.	85.57	0.41	0.26	1.44
PTTG_29960	FtC	Effector	0.96	NADH-ubiquinone oxidoreductase B18 subunit (PF05676)	3.58	7.62	6.18	4.63
PTTG_07625	WTC	Effector	0.956	N.A.	0.00	0.74	35.30	796.90
PTTG_27734	FTC	Effector	0.955	N.A.	0.00	6.66	0.65	1.19
PTTG_26097	FLC	Effector	0.949	N.A.	0.00	16.51	2.41	3.33
PTTG_12486	FGC	Effector	0.949	N.A.	0.72	77.51	8.64	12.86
PTTG_26097	FlC	Effector	0.949	N.A.	0.00	16.51	2.41	3.33
PTTG_29974	FkC	Effector	0.947	N.A.	2.70	1.03	0.71	0.46
PTTG_27769	FlC	Effector	0.946	N.A.	0.55	11.90	2.14	2.36
PTTG_26789	FLC	Effector	0.944	N.A.	0.00	14.19	1.81	2.56

PTTG_26851	YTC	Effector	0.942	N.A.	3.31	0.66	0.42	0.32
PTTG_27529	FQC	Effector	0.94	N.A.	0.22	0.00	0.09	0.08
PTTG_28066	FHC	Effector	0.935	N.A.	0.08	0.63	2.75	13.68
PTTG_30134*	FLC	Effector	0.934	N.A.	0.00	15.64	1.83	2.61
PTTG_25725	FSC	Effector	0.93	N.A.	0.71	20.66	2.68	3.89
PTTG_25944	WVC	Effector	0.925	N.A.	0.10	10.66	4.58	5.19
OAV89437.1	FTC	Effector	0.923	Copper/zinc superoxide dismutase (PF00080)	N.A.	N.A.	N.A.	N.A.
PTTG_25978	FQC	Effector	0.92	N.A.	0.00	14.44	1.56	2.81
PTTG_12285	YPC	Effector	0.918	N.A.	0.53	6.22	1.50	1.91
PTTG_26897	YTC	Effector	0.912	N.A.	0.88	1.45	3.63	6.26
PTTG_26481	YMC	Effector	0.911	N.A.	0.08	0.00	0.00	0.25
PTTG_12428	FLC	Effector	0.909	N.A.	1.36	1.68	2.33	2.23
PTTG_26886	FVC	Effector	0.904	N.A.	0.46	36.42	2.91	4.94
PTTG_12223	FSC	Effector	0.903	N.A.	0.52	69.14	17.04	21.77
PTTG_29256	YRC	Effector	0.902	N.A.	24.83	40.33	28.60	22.67
PTTG_04647	YVC	Effector	0.899	Tuberculosis necrotizing toxin (PF14021)	5.92	0.44	31.83	43.74
PTTG_11693*	FTC	Effector	0.896	N.A.	8847.69	44.60	46.04	164.49
PTTG_11690	FHC	Effector	0.886	DNA damage repair protein Nbs1 (PF08599)	8.94	286.57	43.10	46.43
PTTG_26875	FQC	Effector	0.88	N.A..	0.46	36.42	2.91	4.94
PTTG_27704	FNC	Effector	0.868	N.A.	1181.74	349.34	299.08	264.99
PTTG_12335	YAC	Effector	0.862	Nudix N-terminal (PF14803)	0.00	34.21	4.12	5.44
PTTG_28714	FAC	Effector	0.859	N.A.	1.72	1.59	1.97	2.13
PTTG_28018	FSC	Effector	0.856	N.A.	0.81	0.64	1.82	3.38
PTTG_27646	FAC	Effector	0.855	N.A.	0.00	0.00	0.00	0.03
PTTG_28085	YTC	Effector	0.851	N.A.	5.45	3.45	2.97	2.25
PTTG_27501	FRC	Effector	0.851	Co-chaperone HscB tetracysteine metal binding motif (PF18256)	0.09	0.20	0.60	0.11

PTTG_26059	FFC	Effector	0.851	Cysteine-rich KTR (PF14205)	0.13	0.00	0.14	0.51
OAV88435.1	FAC	Effector	0.846	CFEM domain (PF05730)	N.A.	N.A.	N.A.	N.A.
PTTG_12171*	FgC	Effector	0.846	Saposin A-type domain (PF02199)	6.26	367.81	56.87	46.53
PTTG_27027	YYC	Effector	0.842	N.A.	1.35	5.82	2.98	4.67
PTTG_25982	FSC	Effector	0.841	N.A.	3.23	69.99	357.36	261.70
PTTG_12547	FKC	Effector	0.838	N.A.	0.00	6.70	1.63	4.67
PTTG_12430	FVC	Effector	0.838	N.A.	10.64	117.24	29.78	32.90
PTTG_27318	FTC	Effector	0.837	N.A.	32.13	137.73	24.77	32.99
PTTG_09595	YTC	Effector	0.836	Ring finger domain (PF13639)	0.00	13.57	5.79	6.26
PTTG_25672	FKC	Effector	0.835	N.A.	242.80	6.07	0.61	1.94
PTTG_12548	YTC	Effector	0.83	N.A.	0.00	93.53	13.40	19.22
PTTG_27897	FLC	Effector	0.827	N.A.	0.11	14.59	9.55	13.49
OAV93485.1	FTC	Effector	0.818	Copper/zinc superoxide dismutase (PF00080)	N.A.	N.A.	N.A.	N.A.
PTTG_12508	FDC	Effector	0.815	N.A.	0.00	1.05	0.03	0.08
PTTG_28532	FKC	Effector	0.811	N.A.	0.05	0.13	0.16	0.08
OAV94494.1	FLC	Effector	0.808	Copper/zinc superoxide dismutase (PF00080)	N.A.	N.A.	N.A.	N.A.
PTTG_12290	FlC	Effector	0.8	N.A.	30.80	0.18	0.53	2.78
PTTG_27640	FSC	Effector	0.791	N.A.	0.91	1.58	0.09	0.09
PTTG_29573	FVC	Effector	0.783	Low molecular weight phosphotyrosine protein phosphatase (PF01451)	637.68	80.98	163.16	215.65
PTTG_27737	FPC	Effector	0.78	N.A.	1.52	24.12	26.01	23.04
PTTG_26493	WGC	Effector	0.778	N.A.	0.00	12.56	3.10	7.64
PTTG_25677	WVC	Effector	0.773	N.A.	0.09	0.00	0.25	0.19
PTTG_25643*	FKC	Effector	0.769	N.A.	1190.92	2153.13	116.61	174.89
PTTG_12078	FRC	Effector	0.76	N.A.	0.00	0.83	37.47	112.54
OAV89542.1	FAC	Effector	0.754	Copper/zinc superoxide dismutase (PF00080)	N.A.	N.A.	N.A.	N.A.
novel.470	WKC	Effector	0.746	N.A.	0.00	2.01	0.77	1.13

novel.473	WGC	Effector	0.739	Acyl-protein synthetase LuxE (PF04443)	0.00	3.09	0.39	1.46
PTTG_25377	YIC	Effector	0.733	Protein of unknown function DUF1579 (PF07617)	1162.67	1456.63	1168.90	1103.68
PTTG_10137	YHC	Effector	0.719	Ubiquitin-Binding Zinc Finger (PF18439)	0.09	2.67	2.91	3.31
novel.343	FTC	Effector	0.708	N.A.	0.00	1.73	1.70	1.90
PTTG_25357	WSC	Effector	0.692	N.A.	9.37	3.68	5.36	7.69
PTTG_05870	YKC	Effector	0.677	Protein of unknown function DUF3455 (PF11937)	0.00	6.76	1.39	1.64
PTTG_04698	FIC	Effector	0.677	N.A.	0.05	14.26	12.04	17.38
PTTG_10813	YTC	Effector	0.676	N.A.	0.00	0.00	0.00	0.00
PTTG_12601	FTC	Effector	0.665	N.A.	5.92	0.44	31.83	43.74
PTTG_12311	FGC	Effector	0.663	N.A.	1214.30	9.39	6.13	205.71
PTTG_28678*	FRC	Effector	0.647	N.A.	0.19	0.16	11.11	7.16
PTTG_27148	YYC	Effector	0.64	N.A.	3.69	4.10	36.77	74.96
PTTG_00015	YTC	Effector	0.636	Polysaccharide deacetylase (PF01522)	25.10	2.00	1.97	4.22
PTTG_28615*	FRC	Effector	0.634	N.A.	0.10	0.00	2.72	1.68
PTTG_08463	YRC	Effector	0.629	N.A.	0.18	3.14	1.74	2.39
PTTG_26534	YVC	Effector	0.582	Protein of unknown function DUF3455 (PF11937)	42.84	1.99	34.84	21.65
PTTG_28361	WSC	Effector	0.576	N.A.	14.32	744.44	348.97	444.79
PTTG_11647	FTC	Effector	0.573	N.A.	0.00	9.22	0.94	1.49
PTTG_12313	FHC	Effector	0.567	Protein of unknown function DUF2012 (PF09430)	79.36	63.88	45.04	41.90
PTTG_28678	FrC	Effector	0.647	N.A.	0.19	0.16	11.11	7.16
PTTG_25359	WSC	Non-effector	0.99	N.A.	1.28	1.29	0.48	1.12
PTTG_07660	FLC	Non-effector	0.99	N.A.	0.06	0.25	7.07	7.07
PTTG_03170	FNC	Non-effector	0.99	ABC-2 type transporter (PF01061)<br>ABC transporter (PF00005)	53.85	14.99	20.23	21.23
PTTG_02527	YEC	Non-effector	0.989	Organic solute transporter Ostalpha (PF03619)	83.96	70.03	62.90	54.25
PTTG_01125	FIC	Non-effector	0.989	CFEM domain (PF05730)	375.99	88.83	7.41	12.57

PTTG_28724	FGC	Non-effector	0.988	Alpha galactosidase A (PF16499)<br>Alpha galactosidase C-terminal beta sandwich domain (PF17801)	0.00	20.06	2.05	3.26
PTTG_25440	FAC	Non-effector	0.98	FAD binding domain (PF01565)<br>Berberine and berberine like (PF08031)	3.50	0.30	0.43	1.76
OAV91726.1	YSC	Non-effector	0.97	Hsp70 protein (PF00012)	N.A.	N.A.	N.A.	N.A.
PTTG_01990	YTC	Non-effector	0.957	Polysaccharide deacetylase (PF01522)	1411.39	79.13	104.66	182.52
novel.1172	FSC	Non-effector	0.955	N.A.	0.00	2.98	1.64	1.51
novel.802	YEC	Non-effector	0.937	N.A.	0.00	2.56	1.44	0.98
PTTG_02533	YTC	Non-effector	0.935	Ribosome receptor lysine/proline rich region (PF05104)	0.80	0.40	1.75	4.16
PTTG_07846	WFC	Non-effector	0.918	Dyp-type peroxidase family (PF04261)	280.15	1.98	1.95	32.18
PTTG_26466	YAC	Non-effector	0.914	Jumping translocation breakpoint protein (PF05439)	46.73	116.59	105.29	107.75
PTTG_00013	FNC	Non-effector	0.893	Hepatitis C virus core protein (PF01542)	0.82	1.73	1.86	3.90
PTTG_08384	FVC	Non-effector	0.88	Glycosyl hydrolase family 61 (PF03443)<br>CFEM domain (PF05730)	0.00	11.16	1.77	2.96
PTTG_28635	WLC	Non-effector	0.865	N.A.	0.00	6.53	8.48	7.78
PTTG_04059	FAC	Non-effector	0.795	CFEM domain (PF05730)	1158.27	539.55	275.46	205.49
PTTG_02022	WMC	Non-effector	0.781	N.A.	9.03	0.94	1.59	15.96
PTTG_27779	YPC	Non-effector	0.78	N.A.	0.13	0.00	0.06	0.10
PTTG_25295	FLC	Non-effector	0.78	Protein of unknown function DUF2909 (PF11137)	0.00	31.99	54.90	39.41
PTTG_02013	WMC	Non-effector	0.778	Protein of unknown function DUF1989 (PF09347)	1.94	111.12	415.90	515.52
PTTG_00700	YGC	Non-effector	0.755	Alpha galactosidase A (PF16499)<br>Alpha galactosidase C-terminal beta sandwich domain (PF17801)	1667.96	46.25	72.28	76.16
PTTG_29136	FIC	Non-effector	0.751	Cnl2/NKP2 family protein (PF09447)	0.00	0.35	0.72	0.90
PTTG_08814	FKC	Non-effector	0.734	N.A.	0.00	0.00	0.04	0.00
PTTG_08963	WWC	Non-effector	0.679	Glycosyl hydrolases family 18 (PF00704)	333.53	1.98	5.16	14.40
PTTG_30788	FKC	Non-effector	0.672	Protein of unknown function DUF3836 (PF12930)	0.08	0.00	0.15	0.12

PTTG_28949	FVC	Non-effector	0.668	CFEM domain (PF05730)	0.06	0.75	0.76	0.86
PTTG_28156	WCC	Non-effector	0.65	N.A.	0.00	9.26	16.91	20.96
PTTG_27608	WAC	Non-effector	0.64	N.A.	34.04	76.61	169.70	130.81
PTTG_27798	FFC	Non-effector	0.636	N.A.	182.40	54.87	68.75	69.05
PTTG_08285	YPC	Non-effector	0.635	Follistatin/Osteonectin-like EGF domain (PF09289)	2.91	7.43	18.19	32.30
PTTG_05502	WGC	Non-effector	0.615	N.A.	9.02	4.57	3.78	4.58
PTTG_26417	FCC	Non-effector	0.61	N.A.	1.54	3.44	5.00	5.66
PTTG_29231	YSC	Non-effector	0.589	N.A.	15.75	11.87	10.93	11.14
PTTG_25926	FDC	Non-effector	0.582	N.A.	0.00	0.00	0.00	0.00
PTTG_00613	FEC	Non-effector	0.58	N.A.	566.59	88.65	154.11	172.90
PTTG_28636	WLC	Non-effector	0.557	N.A.	0.00	1.52	2.56	1.91
PTTG_00286	WGC	Non-effector	0.535	N.A.	22.70	16.58	32.72	31.23
novel.1931	WLC	Non-effector	0.527	ABC transporter (PF00005)	0.04	0.64	0.87	1.03
novel.540	FHC	Non-effector	0.518	N.A.	0.00	0.05	0.13	0.11
PTTG_26761	FNC	Non-effector	0.504	WD domain, G-beta repeat (PF00400)	0.00	0.00	0.00	0.00
PTTG_28319	FPC	Non-effector	0.503	gag-polypeptide of LTR copia-type (PF14223)<br>Zinc knuckle (PF00098)	3.32	2.04	2.19	1.72
PTTG_09706	YTC	Unlikely effector	0.536	N.A.	0.00	98.73	10.41	16.64
PTTG_11739*	FKC	Unlikely effector	0.52	N.A.	1.97	502.64	31.80	48.26

* Effector candidates selected for gene cloning and initial functional characterization.
